# Reduced trolling on Russian holidays and daily US Presidential election odds

**DOI:** 10.1371/journal.pone.0264507

**Published:** 2022-03-30

**Authors:** Douglas Almond, Xinming Du, Alana Vogel

**Affiliations:** 1 Columbia University, New York, NY, United States of America; 2 National Bureau of Economic Research, Cambridge, MA, United States of America; University of Oxford, UNITED KINGDOM

## Abstract

Russian trolls generally supported the Trump campaign and were particularly active on Twitter 2015-2017. We find that trolling fell 35% on Russian holidays and to a lesser extent, when temperatures were cold in St. Petersburg. Exogenous variation in trolling by day allows us to consider indirectly-affected political behaviors in the US—outcomes that are less traceable via tweet sharing but potentially more important to policymakers than the direct dissemination previously studied. As a case in point, we describe reduced form evidence that Russian holidays affected daily trading prices in 2016 election betting markets. This response is consistent with successful Russian interference in support of Trump.

## Introduction

Professional Russian trolling is malevolent and murky. The February 2018 US grand-jury indictment states that the Internet Research Agency (IRA) in St. Petersburg conducted “information warfare” against the United State by using “fictitious U.S. personas on social media platforms….” (US Department of Justice, 2018). These fake personas communicated with unwitting members of the public to sow distrust in the US political system, discourage minorities from voting, make assertions of voter fraud, organize political rallies, stoke racial divisions (Senate Intelligence Committee, 2019), and other illicit activities (US Department of Justice, 2018). According to the Senate Intelligence Committee’s bipartisan report, Russia interfered to assist the Trump campaign:

*“The Committee found that the IRA sought to influence the 2016 U.S. presidential election by harming Hillary Clinton’s chances of success and supporting Donald Trump at the direction of the Kremlin. The Committee found that IRA social media activity was overtly and almost invariably supportive of then-candidate Trump to the detriment of Secretary Clinton’s campaign.”*

IRA employees used an Internet proxy service to conceal their I.P. addresses [1] and “covering tracks” was a goal of the IRA operation (US Department of Justice, 2018). The operation’s stealth has made it it difficult to know what—if any—its impacts were. This is particularly true for “downstream” outcomes: those where the precise path of content re-tweeting, sharing, etc. and its behavioral effects cannot be traced explicitly. As has been widely reported, Russian interference is believed by many to have continued during the 2020 election cycle.

Empirically, Russian trolling activity is correlated with the timing of other legitimate activities, including breaking news stories. For example, Russian troll accounts “respond to shifts in political circumstances,” e.g. “the well-known faint/stumble by Hillary Clinton leaving a 9/11 commemoration event, followed by her pausing the campaign with an announcement of pneumonia,” [2] and overall internet traffic levels. Additionally, trolling activity exhibits time trends, seasonality, and the influence of the day of the week. This can make it difficult to ascribe time-series variation in trolling intensity and its effects to the work of trolls *per se*, as opposed to other factors.

Here we explore factors behind Russian trolling activity in the US that are likely specific to Russia. This forensic exercise [3] may be useful for shedding light on the production function of Russian trolling. To the extent they are indeed coordinated in Russia, this would be consistent with Twitter’s identification of particular English-language tweets as of surreptitiously Russian-origin (see Section ‘Russian Interference via Twitter’). Most important for future research, our approach might allow researchers to isolate exogenous drivers of trolling activity in the US—neglected by research to date—and thereby its true and full effect. (We use “exogenous” to imply the absence of a correlation between an explanatory variable and the error term. That is, we think Russian holidays are uncorrelated with other unobserved determinants of outcomes we analyze, and thereby constitute a natural experiment in Russian trolling intensity).

## Major findings

We find that Russian holidays decrease trolling by 35% in the corpus of Twitter’s primary data release on October 2018 of 2.9 IRA million tweets. The decrease in overall holiday tweeting is largest for original tweets, which may be more impactful than retweets. To a lesser extent, warmer temperatures in St. Petersburg also increased trolling.

Despite both popular interest and its implications for national security, remarkably little is known about the causal effects of Russian trolling on outcomes politicians and policymakers actually care about, such as election prospects. We argue below that exogenous variation in Russian trolling could be useful in forging a link—albeit it indirect—to these more important outcomes. Such indirect effects would include the downstream impact of Russian trolls on unwitting internet users who do not retweet the (surreptitiously) Russian tweet or its content and how they and their US compatriots behave when off Twitter. Arguably, these outcomes are more important than the direct observed dissemination of troll content itself that has previously been studied.

When interference drops markedly on Russian holidays, we find a corresponding response in Presidential election odds as inferred from online betting markets. Consistent with the consensus view that Russian interference sought to hurt the Democratic opponents’ chances, Democrats’ odds peak on Russian holidays (and Republican odds hit their nadir). To our knowledge, this is the most comprehensive Russian election interference impact documented empirically for the US.

## Related research

Previous research has focused on persons who had direct interactions with Russian trolls and automated bots. Importantly, this direct dissemination can and has been observed on social media platforms, where the interference itself originates.

[4] surveyed Twitter users in the fall of 2017 and observed in longitudinal data whether they directly accessed content from IRA accounts, the true IRA identities of which had not been disclosed to the public at that time. [4] note that prior to their paper:

*Yet, to our knowledge, no studies have examined whether these efforts actually impacted the attitudes and behaviors of the American public*.

At follow-up, [4] found “no evidence that interacting with these accounts substantially impacted 6 political attitudes and behaviors.” They reason that IRA activity may not show its intended effect because it was accessed disproportionately by (US) Twitter users who were already polarized. That is, there is selection into who saw the IRA content that confounds estimates of IRA impacts.

In contrast, a working paper by [5] analyzes Twitter behavior of those users who were actively contacted by automated IRA bots and provide “some of the first evidence that contacted Twitter users’ behavior underwent significant changes…following interactions with Russia’s Internet Research Agency.” Still, they characterize their working paper’s findings of changes in tweeting frequency and tweet sentiment as correlational: “The intent of this paper is not to establish causality” [5]. Focussing on (automated) Russian bots, [6] find that “diffusion of information on Twitter is largely complete within 1–2 hours” and have a “tangible effect on the tweeting activity of humans”. A working paper by [7] builds a machine learning algorithm to predict the likelihood that an account is a Russian troll, unbeknownst to other Twitter users. They then see to what extent these likely-troll accounts diffuse content to journalists. Anecdotally, the Washington Post noted that Eric Trump, Donald Trump Jr., and Kellyanne Conway all posted information from a likely Russian troll @TEN_GOP in the run-up to the 2016 presidential election.

For social media content more broadly, causal effects for “downstream” political outcomes like street protests or voter turnout have been shown using natural experiments, e.g. *within* Russia itself [8]. In terms of content dissemination via social media, [9] note: “As immediate reactions are often based on emotions rather than reason, fake news, which evokes fear or anger, may spread faster than real news, which is often less emotionally charged.”

For Russian trolling that targets Americans, we know of no natural experiment-based evidence that it affected the more important downstream outcomes that are not directly linkable with tweet- or account-level trolling data. Here, we describe new evidence that Russian holidays: 1) markedly reduced trolling activities, and; 2) impacted daily trading prices in 2016 election betting markets.

## Methods and research design

### Data

#### Russian interference via Twitter

According to the federal indictment, the IRA began its interference in the US around 2014 with its English-language “translator project” (US Department of Justice, 2018). [4] cite a US Senate Intelligence Committee that IRA has been active since 2013. Twitter blocked suspicious tweets posted back to 2009. This English-language group was “elite and secretive” [1]. In July 2016, more than eighty IRA employees were assigned to this covert effort.

In October 2018, Twitter released 2.9 million English-language tweets from 3,841 accounts as “affiliated with the IRA”, which we will refer to as “Wave 1” tweets. What caused Twitter’s suspicions of particular accounts and tweets they have flagged was not disclosed. (In June 2019, Twitter stated: “…we employ a range of open-source and proprietary signals and tools to identify when attempted coordinated manipulation may be taking place, as well as the actors responsible for it.”) Twitter blocking occurs *after* a substantial period of unchecked online activity, which is what we analyze. In its October 2018 announcement, Twitter wrote:

*[W]e are releasing the full, comprehensive archives of the Tweets and media that are connected with these two previously disclosed and potentially state-backed operations on our service. We are making this data available with the goal of encouraging open research and investigation of these behaviors from researchers and academics around the world. These large datasets comprise 3,841 accounts affiliated with the IRA, originating in Russia, and 770 other accounts, potentially originating in Iran. They include more than 10 million Tweets and more than 2 million images, GIFs, videos, and Periscope broadcasts…*.

Since the October 2018 release, Twitter has continued detecting and suspending accounts with known state-backed information operations. As of October 2020, Twitter released 418, 4 and 1,152 blocked accounts linked with Russia in January 2019, June 2019 and June 2020, respectively, totaling roughly 770,000 English-language tweets. We refer to these releases as “waves” 2–4.

Unfortunately, the date/time at which the individual account was blocked or removed is not disclosed by Twitter (see S4 section in S1 File for an attempt at analyzing inferred blocking dates. We thank Joe Doyle for a conversation that spurred this RD-style investigation). Nor do we know whether the suspect accounts disclosed in the same blocking announcement were blocked simultaneously or separately. We focus on English tweets on the “day shift”: 9am-9pm Russia time. [1] notes that IRA work shifts began at 9am and finished at 9pm. [2] likewise note: “IRA employees tasked with making social media posts are reported to have been organized into 12-hour shifts, a day shift and night shift, and instructed to make posts at times appropriate to U.S. time zones.”

Turning to trolling content, [2] document “enormous heterogeneity in theme and approach across IRA accounts”. For example, some tweets appear targeted at right-wing followers and others to sow discord on the left. There is also substantial variation over time in trolling intensity: the time series by wave is shown in S1 Fig in S1 File. We see very little (subsequently-unmasked) trolling activity after the fall of 2018. Also, the vast majority of English tweets—some 80%—come from wave 1, which were most active from late 2014 through the end of 2017.

#### Downstream outcomes

According to the February 2018 indictment, IRA sought to develop “certain fictitious U.S. personas into “leader[s] of public opinion in the United States” (US Department of Justice, 2018). Therefore, we look at the Hedonometer, a summary, widely-referenced metric of “average happiness” of English tweets on Twitter. We obtained the publicly-available time series at the daily level, developed by [10]. (The Hedonometer data is available online: http://hedonometer.org/index.html) The data extend back September 2008 through the present. For individual words, the scale of perceived happiness score is 1 to 9. In the daily aggregate measure, Hedonometer ranges between 5 and 7.

Hedonometer notes on its website: “Our Hedonometer is based on people’s online expressions, capitalizing on data-rich social media, and we’re measuring how people present themselves to the outside world”. [10] provide detailed information on their scoring algorithm. An advantage to analyzing these downstream data is that they are available for *all* the initial posting dates of the subsequently-blocked accounts and tweets. As only 1,702 tweets posted before 2012 were subsequently blocked, we use 2012 as the start year of our study period. If Russian trolling had broad impacts on *general* perceptions of society and politics in the US, we hypothesize it can be captured by this summary metric of sentiment.

Turning to political economy outcomes, [11] note there have been “large and well-organized markets for betting on presidential elections” stretching back to at least 1868. 2020 Election odds come from BetData, which tracks odds for 105 potential candidates beginning in November 2016. Because the identified trolls were most active before the November 2016 election, we have less overlap between our election odds data and observed troll activity. 2016 Presidential election betting data come from Iowa Election Markets (IEM) and PredictIt, which both begin in November 2014. Since we observe daily prices for 2016 Election odds and hourly prices for 2020 Election odds, we use the daily closing price for 2016 election odds and hourly price at 2pm EST (9pm Russian time) for 2020 odds. We use the implied probability of winning for Republican’s and Democrat’s candidates as outcome variables.

A key difference of these markets is that PredictIt only allows traders in the US, BetData (Betfair) forbids US traders (from Betfair’s website: “You will not bet or attempt to bet with us if you are located in the United States of America or in any other country with a comparable legal situation. These countries will be determined by Betfair from time to time.”) IEM is open to traders worldwide (from the IEM Trader’s Manual: “Participation in the IEM is open to students, faculty, and staff at colleges and universities worldwide; IEM political markets are also open to non-academic participants”).

### Research design

#### Russian holidays

We define Russian holidays using the eight Federal holidays enumerated within the Labor Code of the Russian Federation. The eight Russian holidays are:

New Years (January 1)
Eastern Orthodox Christmas (January 7)
Defender of the Fatherland Day (February 23)
International Women’s Day (March 8)
Labor Day (May 1)
Victory Day (May 9)
Russia Day (June 12)
Unity Day (November 4).

We do not use January 1 because it coincides with a US holiday, which we will also control for. We also explored re-assigning holidays that fall on weekends to their additional celebration on adjoining workdays, but this did not strengthen our first stage relationship. (The initial version of our manuscript incorrectly assigned Defender of the Fatherland holiday as February 25. Correcting this error yields stronger effects).

Because the whole January 1–7 week may be a holiday, we add robustness checks in S7 Table in S1 File. Including January 2–6 as holidays still generates significant holiday effects but the magnitude falls. In the event study setting, dropping these two holidays generates larger holiday impacts in both #tweets and downstream outcomes. This suggests our holiday story for reduced trolling activity does not extend to January 2–6, and is more striking (and remains statistically significant) for the six holidays after January.

We are not the first to notice a relationship between Russian holidays and Russian malfeasance in the US. In 2015, a cybersecurity firm in California was studying “an advanced persistent threat group that we suspect the Russian government sponsors”, which they referred to as APT29. Their focus was not on Russian trolling by the IRA, but instead non-public actions by Russian intelligence itself. Released in July 2015, the “Hamertoss” threat intelligence report by Fireeye Inc. noted in passing that “APT29 appeared to cease operations on Russian holidays….”. They did not present any additional details or empirical evidence. To our knowledge, this holiday effect has not been picked up in the academic literature studying Russian interference. And fortunately for our approach, Russian holidays differ from US holidays, which may have distinct effects on outcomes of interest.

#### Ambient temperature

Daily temperature data is from the National Climatic Data Center Global Summary of the Day (GSOD) dataset: https://www.ncei.noaa.gov/access/search/data-search/global-summary-of-the-day.

We use mean temperature from weather stations in St. Petersburg 2012–2019 for the main result, and add London and other cities in U.S. in S1.3 Section in S1 File.

#### Wave-specific factors

S1 Fig in S1 File shows that the level of tweeting differs markedly by wave of release. Additionally, Wave 1 was specifically attributed to the Internet Research Agency, while subsequent waves of blocked Russian tweets were not. Trolling tactics and practices evolved over time and it is possible that different groups of actors in differing locations within Russia were involved in different waves. For these reasons, our regression specifications include wave-specific fixed effects and allow the effect of temperature and holiday to vary by wave:

$$\begin{array}{c} \begin{array}{c} Y_{wt}=\sum_{w=1}^{4}\beta_{w}Holiday\_RU_{t}\times Wave_{w}+\sum_{w=1}^{4}\gamma_{w}Temperature_{t}\times Wave_{w}\\ +\theta_{t}Holiday\_US_{t}+DOW_{t}+{\left(Wave*Year\right)}_{wt}+{\left(Wave*Month\right)}_{wt}+\varepsilon_{wt} \end{array} \end{array}$$ (1)

where Y_wt_ denotes the number of wave *w* tweets posted 9am-9pm Russian time on day *t*. We drop tweets with blank tweet_text for further analysis. Among non-blank tweets, we consider pure retweets without comments as retweets, non-retweets or retweets with comments as original tweets. In practice, we label tweet as retweet if it starts with “RT” and its field is_retweet is true. In S6 Section in S1 File, we add back blank tweets and only use field is_retweet to label retweets. Results are very similar to main results without blank tweets.

Coefficients *β*_1_ to *β*_4_ capture wave-specific impacts of Russian holidays, and *γ*_1_ to *γ*_4_ capture temperature impacts. We also include a Holiday_US_t_ dummy and day of week fixed effects that are common across waves. (Wave*Year)_*wt*_ and (Wave*Month)_*wt*_ denote year and month FE that are interacted with the wave dummies.

## Impact of Russian holidays and temperature on trolling volume

### Wave by day results

In Table 1, we analyze how Russian holidays and temperature in St. Petersburg affect the total number of suspect (i.e. subsequently-blocked) tweets at the wave-day level. To isolate the holiday effect, we control for day-of-week fixed effects (FE), wave by year FE, and wave-specific seasonality (wave by calendar month FE). The number of blocked tweets released in the first wave during Russian holidays decreases by 0.34 standard deviations or 41.2% relative to the mean, as compared with non-holidays. The 0.34 point estimate is fairly precise, with a standard error of.09 to.10. Also on the first wave, a one standard deviation increase in temperature more modestly increases blocked tweets by 0.056 standard deviations, or 6.8% relative to the mean. Holiday and temperature estimates appear driven by the subset of original tweets (Panel B), which might be expected to have a greater impact on downstream outcomes than retweets (Panel C).

**Table 1:** First stage: Russian holiday and temperature on blocked tweets on the day shift.

	Panel A: #All tweets (z)					
	(1)	(2)	(3)	(4)	(5)	(6)
Holiday_RU × Wave = 1	-0.339***	-0.338***	-0.338***	-0.338***	-0.340***	-0.340***
	(0.103)	(0.103)	(0.103)	(0.103)	(0.092)	(0.093)
Holiday_RU × Wave = 2	-0.040	-0.039	-0.039	-0.039	-0.039	-0.039
	(0.103)	(0.103)	(0.103)	(0.103)	(0.092)	(0.093)
Holiday_RU × Wave = 3	-0.005	-0.004	-0.004	-0.004	-0.005	-0.005
	(0.103)	(0.103)	(0.103)	(0.103)	(0.092)	(0.093)
Holiday_RU × Wave = 4	-0.007	-0.006	-0.006	-0.006	-0.007	-0.007
	(0.103)	(0.103)	(0.103)	(0.103)	(0.092)	(0.093)
Temperature × Wave = 1 (z)	.0566*	.0566*	.0557*	.0546*	.00118	.00113
	(.0323)	(.0323)	(.0323)	(.0323)	(.0332)	(.0332)
Temperature × Wave = 2 (z)	-.0148	-.0148	-.0157	-.0168	.00443	.00437
	(.0323)	(.0323)	(.0323)	(.0323)	(.0332)	(.0332)
Temperature × Wave = 3 (z)	.00114	.00113	.00021	-.000872	.00169	.00164
	(.0323)	(.0323)	(.0323)	(.0323)	(.0332)	(.0332)
Temperature × Wave = 4 (z)	-.00088	-.000883	-.00181	-.00289	.000345	.000291
	(.0323)	(.0323)	(.0323)	(.0323)	(.0332)	(.0332)
Holiday_US	.0128	.0129	.0127	.0128	.0138	.0138
	(.0474)	(.0475)	(.0474)	(.0474)	(.0426)	(.0426)
Days (×10^−3^)		.0924	.617	.853		-.047
		(.854)	(.861)	(.876)		(2.28)
Days^2^ (×10^−6^)			-.186***	-.428**		.308
			(.0421)	(.172)		(1.8)
Days^3^ (×10^−9^)				.0553		-.103
				(.0382)		(.398)
Observations	11560	11560	11560	11560	11560	11560
R-square	0.363	0.363	0.364	0.364	0.500	0.500
	Panel B: #Original tweets (z)					
Holiday_RU × Wave = 1	-0.315***	-0.314***	-0.314***	-0.314***	-0.316***	-0.316***
	(0.109)	(0.109)	(0.109)	(0.109)	(0.100)	(0.100)
Holiday_RU × Wave = 2	-0.023	-0.023	-0.023	-0.023	-0.023	-0.023
	(0.109)	(0.109)	(0.109)	(0.109)	(0.100)	(0.100)
Holiday_RU × Wave = 3	-0.005	-0.005	-0.005	-0.004	-0.005	-0.005
	(0.109)	(0.109)	(0.109)	(0.109)	(0.100)	(0.100)
Holiday_RU × Wave = 4	-0.007	-0.007	-0.006	-0.006	-0.008	-0.007
	(0.109)	(0.109)	(0.109)	(0.109)	(0.100)	(0.100)
Temperature × Wave = 1 (z)	.0959***	.0959***	.0953***	.0918***	.0296	.0296
	(.0342)	(.0342)	(.0342)	(.0342)	(.0358)	(.0358)
Temperature × Wave = 2 (z)	.0062	.0062	.00561	.00216	.00943	.0094
	(.0342)	(.0342)	(.0342)	(.0342)	(.0358)	(.0358)
Temperature × Wave = 3 (z)	.000528	.000525	-.0000635	-.00351	.000901	.000876
	(.0342)	(.0342)	(.0342)	(.0342)	(.0358)	(.0358)
Temperature × Wave = 4 (z)	-.00183	-.00183	-.00242	-.00586	-.000772	-.000797
	(.0342)	(.0342)	(.0342)	(.0342)	(.0358)	(.0358)
Holiday_US	.0278	.0279	.0278	.0283	.0301	.0301
	(.0502)	(.0502)	(.0502)	(.0502)	(.046)	(.046)
Days (×10^−3^)		.0851	.42	1.17		.0963
		(.904)	(.912)	(.927)		(2.46)
Days^2^ (×10^−6^)			-.119***	-.889***		.125
			(.0446)	(.182)		(1.94)
Days^3^ (×10^−9^)				.176***		-.0514
				(.0404)		(.43)
Observations	11560	11560	11560	11560	11560	11560
R-square	0.283	0.283	0.284	0.285	0.415	0.415
	Panel C: #Retweeted tweets (z)					
Holiday_RU × Wave = 1	-0.194*	-0.193*	-0.193*	-0.193*	-0.195**	-0.195**
	(0.103)	(0.103)	(0.103)	(0.102)	(0.085)	(0.085)
Holiday_RU × Wave = 2	-0.049	-0.049	-0.049	-0.049	-0.048	-0.048
	(0.103)	(0.103)	(0.103)	(0.102)	(0.085)	(0.085)
Holiday_RU × Wave = 3	-0.001	-0.000	0.000	-0.000	-0.000	-0.001
	(0.103)	(0.103)	(0.103)	(0.102)	(0.085)	(0.085)
Holiday_RU × Wave = 4	-0.002	-0.002	-0.001	-0.002	-0.002	-0.002
	(0.103)	(0.103)	(0.103)	(0.102)	(0.085)	(0.085)
Temperature × Wave = 1 (z)	-.0534*	-.0534*	-.0544*	-.0502	-.0559*	-.056*
	(.0322)	(.0322)	(.0321)	(.0321)	(.0304)	(.0304)
Temperature × Wave = 2 (z)	-.0481	-.0481	-.0491	-.0449	-.00801	-.00809
	(.0322)	(.0322)	(.0321)	(.0321)	(.0304)	(.0304)
Temperature × Wave = 3 (z)	.0017	.0017	.000635	.00486	.00231	.00223
	(.0322)	(.0322)	(.0321)	(.0321)	(.0304)	(.0304)
Temperature × Wave = 4 (z)	.0015	.00149	.00043	.00465	.00237	.00229
	(.0322)	(.0322)	(.0321)	(.0321)	(.0304)	(.0304)
Holiday_US	-.0243	-.0242	-.0245	-.0252	-.0264	-.0265
	(.0472)	(.0472)	(.0472)	(.0471)	(.039)	(.039)
Days (×10^−3^)		.0543	.659	-.26		-.305
		(.85)	(.857)	(.871)		(2.08)
Days^2^ (×10^−6^)			-.214***	.73***		.496
			(.0419)	(.171)		(1.65)
Days^3^ (×10^−9^)				-.216***		-.147
				(.038)		(.364)
Observations	11560	11560	11560	11560	11560	11560
R-square	0.373	0.373	0.374	0.376	0.584	0.584
DOW FEs	Y	Y	Y	Y	Y	Y
Wave-Month FEs	Y	Y	Y	Y		
Wave-Year FEs	Y	Y	Y	Y		
Wave-Year-Month FEs					Y	Y

*Notes*: The smaller sample size than four times #days 2012–2019 is due to 32 days with no temperature data.

* significant 10% level;

** significant at 5% level;

*** significant at 1% level.

In Columns (2)-(4), we add linear, quadratic, and cubic day trends in the regression to allow for the nonlinear time trend in Russian trolling activity. Since we already control for wave by year FE, we assume the same within-year trends for the four waves after controlling wave-specific seasonality. R^2^s are similar in Columns (1)-(4), indicating a poor explanatory contribution from the time trends. Coefficient estimates on holidays and temperature remain stable with these additional controls. In Columns (5) and (6), we conduct a more aggressive control strategy, leveraging within year-by-month-by-wave comparisons. Adding these fixed effects increases the R^2^ from.36 to.5 (R^2^s are higher for the retweeting operations than original tweets). The impact estimate for Russian holidays is highly robust, the point estimate remaining at -.34 standard deviations. Though estimates on temperature are not significant, we think seasonality of temperature in St. Petersburg is sufficiently controlled with calendar month FE and we will only use Column (1)-(4) estimates to gauge the impact of local temperature fluctuations. Adding year-month FE may absorb temperature fluctuations that last for several days and are registered by the year by month FE.

In contrast to the first wave, the impact of holiday or temperature on tweets released in other three waves is not significantly different from 0. As mentioned above, the latter three releases together contain 20% of the total English-language troll tweets Twitter has tied to Russia to date.

### Wave 1 event study

**Fig 1:**
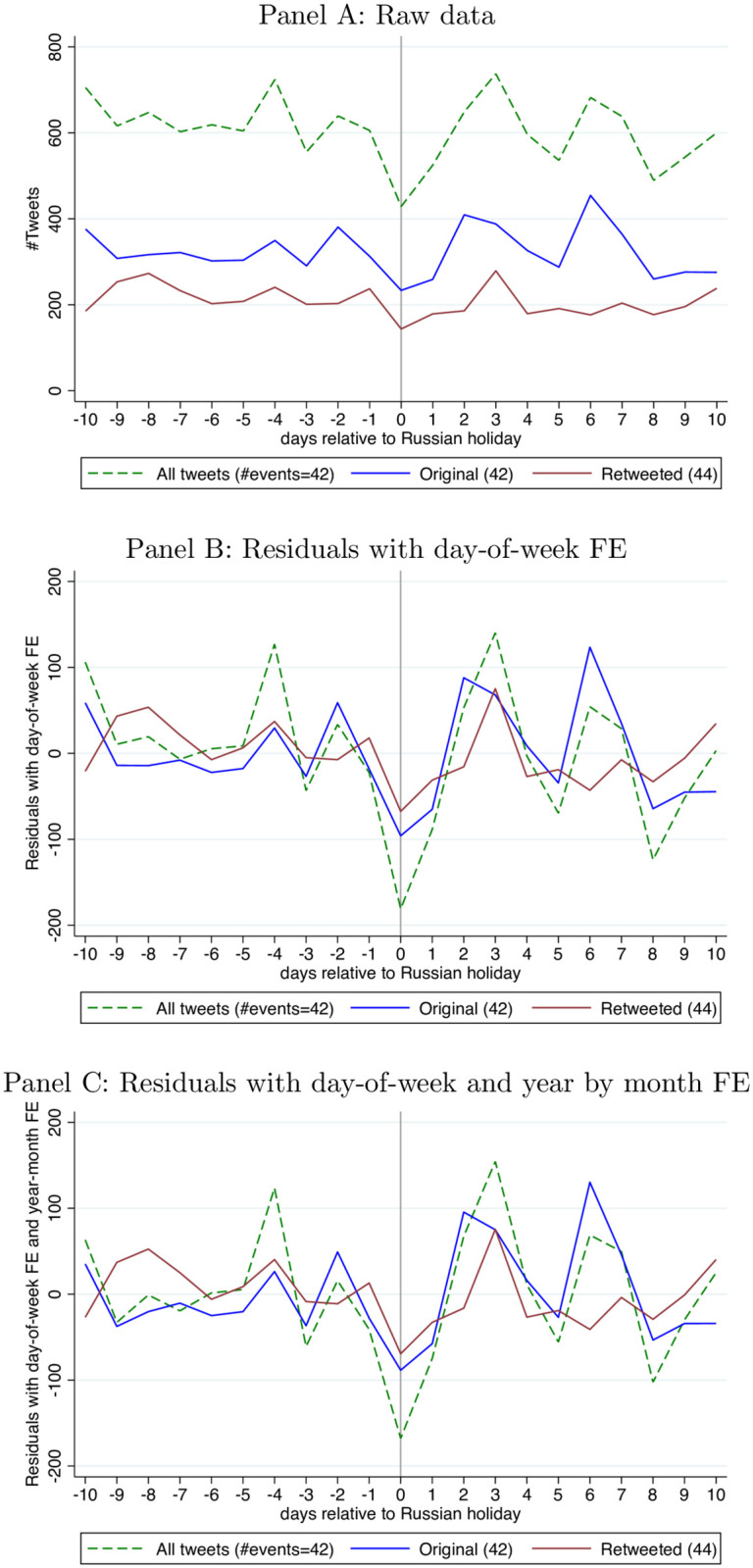
Event study of wave 1 tweets (day shift) around Russian holidays 2012–2017. (We drop top 10 busiest days for each category (all, original, retweeted tweets) 2012–2017, and only keep holiday events with complete data over the 21-day window. This results in a smaller number of events than 48 (8 events per year over 6 years) and a different number of events for each category, reported in parentheses. For Panel A, we calculate the simple average of tweets on each event day. For Panel B and C, we add day-of-week FE and year-month FE and calculate the average of predicted residuals). Panel A: Raw data. Panel B: Residuals with day-of-week FE. Panel C: Residuals with day-of-week and year by month FE.

We show the holiday impact on the first wave “mother lode” of tweets in Fig 1. Over the 2012–2017 activity period for wave 1 accounts, we use 42 holidays for original tweets and 44 holidays for retweets. Without any control variables, the number of tweets decreases by roughly 200 on day 0 and the magnitude is very similar to our point estimates in Table 1 Panel A, which includes a more extensive set of regression controls. In the event that holidays coincide with weekends or are otherwise correlated with the day of the week, we add day-of-week FE (only, no other control variables) in the middle panel. The pattern is quite similar and the trough on day 0 (the holiday itself) is more obvious.

In addition to day of the week FE, S8 Table in S1 File adds additional control variables and shows that the holiday effect is robust and statistically significant. These regression controls include year and month FE, and even year by month FE, thereby restricting comparisons to be within the same month. The bottom panel of Fig 1 displays the same-month comparisons. Residuals with day-of-week and year-month FE decrease by 90 original tweets and 80 retweets on day 0.

**Fig 2:**
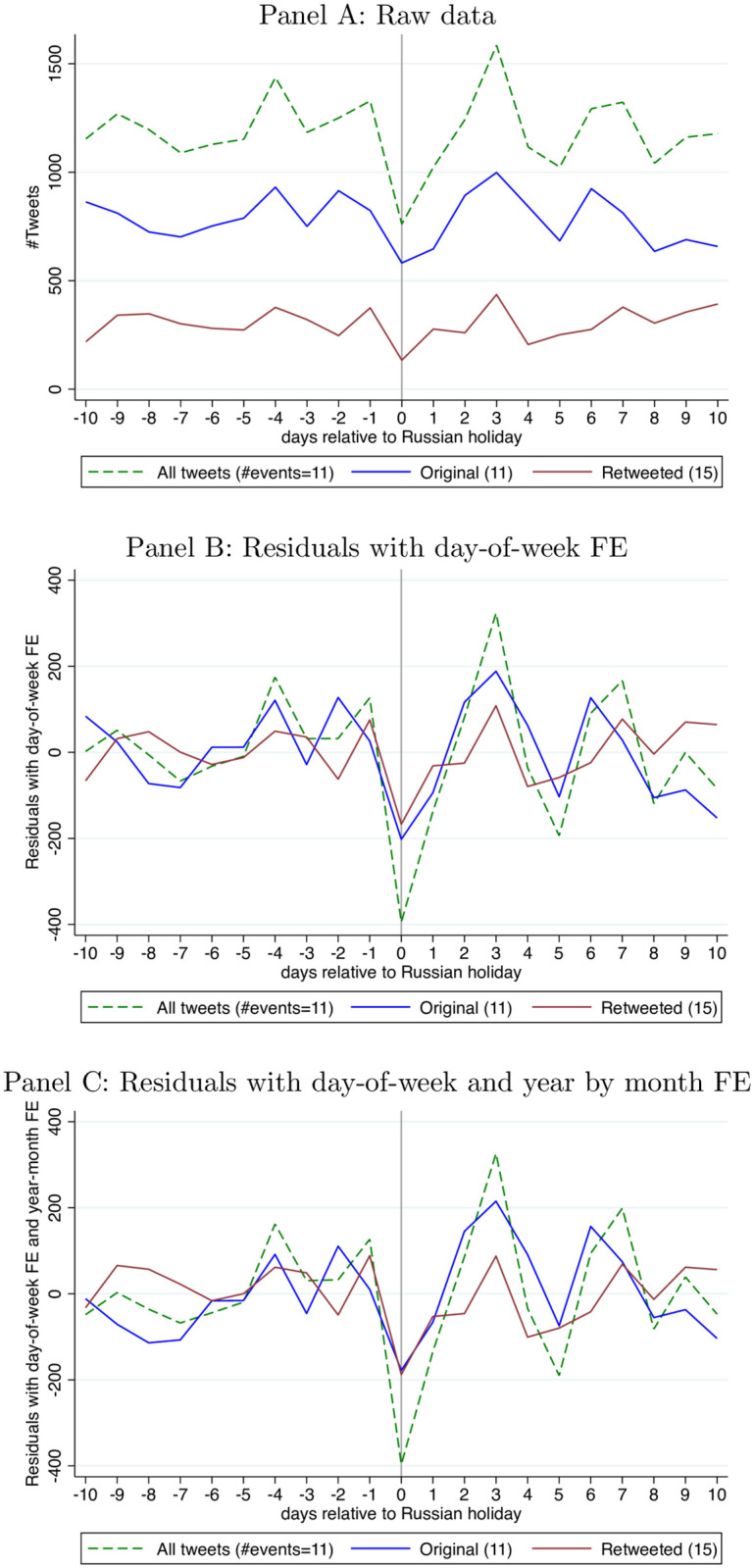
Wave 1 tweets (day shift) around Russian holidays Nov. 2014–Nov. 2016. (Similar to Fig 1, we drop top 10 busiest days for each category (all, original, retweeted tweets) 2012–2017, and only keep holiday events with complete data over the 21-day window. This results in a smaller number of events than 15 (8 events per year over 2 years, minus Unity Day (Nov 4) 2016 with incomplete betting data) and different number of events for each category, reported in parentheses). Panel A: Raw data. Panel B: Residuals with day-of-week FE. Panel C: Residuals with day-of-week and year by month FE.

Fig 2 repeats this analysis but focussing on the sub-period of the 2016 election campaign: November 2014-November 2016. The holiday pattern is if anything more pronounced during these two years, this despite using only 11 holidays for original tweets and 15 holidays for retweets. Again, this pattern is highly robust to regression controls.

### Pre-holiday trends and robustness

Visually, we do not see systematic pre-holiday trends in the event-study figures. In S10 Table in S1 File, we consider statistically whether days away from Russian holidays are associated with increases or decreases in trolling. This tabular analysis confirms the absence of pre-holiday trends in trolling activity and that the outlier is the holiday itself.

First stage estimates are robust when we drop the top 10 busiest days for each wave 2012–2019 (S1.2 Section in S1 File). The magnitude of the holiday effect is somewhat smaller than that in Table 1. Although very busy trolling days contribute some to the estimated holiday effect, we still see a qualitatively-similar decrease in the number of tweets on holidays as compared with the non-busiest days (busiest days may also be an outcome of the Russian holiday schedule). Dropping the temperature in St. Petersburg variable, i.e. only focussing on holidays, also yields similar first stage estimates (S1.1 Section in S1 File). We also control for daily temperature in other major American/English cities: London, New York, Los Angeles, and Washington DC (S1.3 Section in S1 File). The first stage point estimates have slightly larger magnitudes with the inclusion of temperature controls in the four Western cities.

### First stage mechanism

Holiday timing is an intuitive and common natural experiment in economics. For example, avoidance by physicians of holiday deliveries creates a pronounced dip in induced deliveries, which can be used to evaluate health impacts of induction on surrounding days [12]. Part of the reason is that the shadow cost of leisure increases markedly on holidays. This is a fairly straight-forward mechanism for reduced Russian trolling activity on Russian holidays.

Turning to temperature, St. Petersburg is cold. In July, the average daily high temperature is 72 °F and daily high temperatures in January are typically below freezing. IRA work has been characterized as occurring indoors and concentrated for a time at an office building at 55 Savushkina Street in St. Petersburg. In an office environment, task productivity peaks around 72 °F [13, 14]. To the extent that indoor climate control is imperfect at trolling workplaces, warmer ambient temperatures may bring the indoor temperature closer to the productivity optimum. Alternatively, it could be that cold experienced outside of work has a persistent effect on productivity while at work. Finally, colder temperatures may be associated with factors like ice that make it more difficult to get to work.

For completeness, we note factors that did not appear to affect trolling activity. Domestic Russian protests may have distracted IRA workers from the US-interference operation. Motivated by the observation that “employees were mostly in their 20s” [1], we also considered the schedule of local hockey games and Russian national football team games. None appeared to affect the intensity of English-language trolling activity. Finally, increased Brexit trolling did not appear to decrease non-Brexit trolling (through a trolling supply constraint).

## Impact of Russian factors on election odds and hedonometer

**Fig 3:**
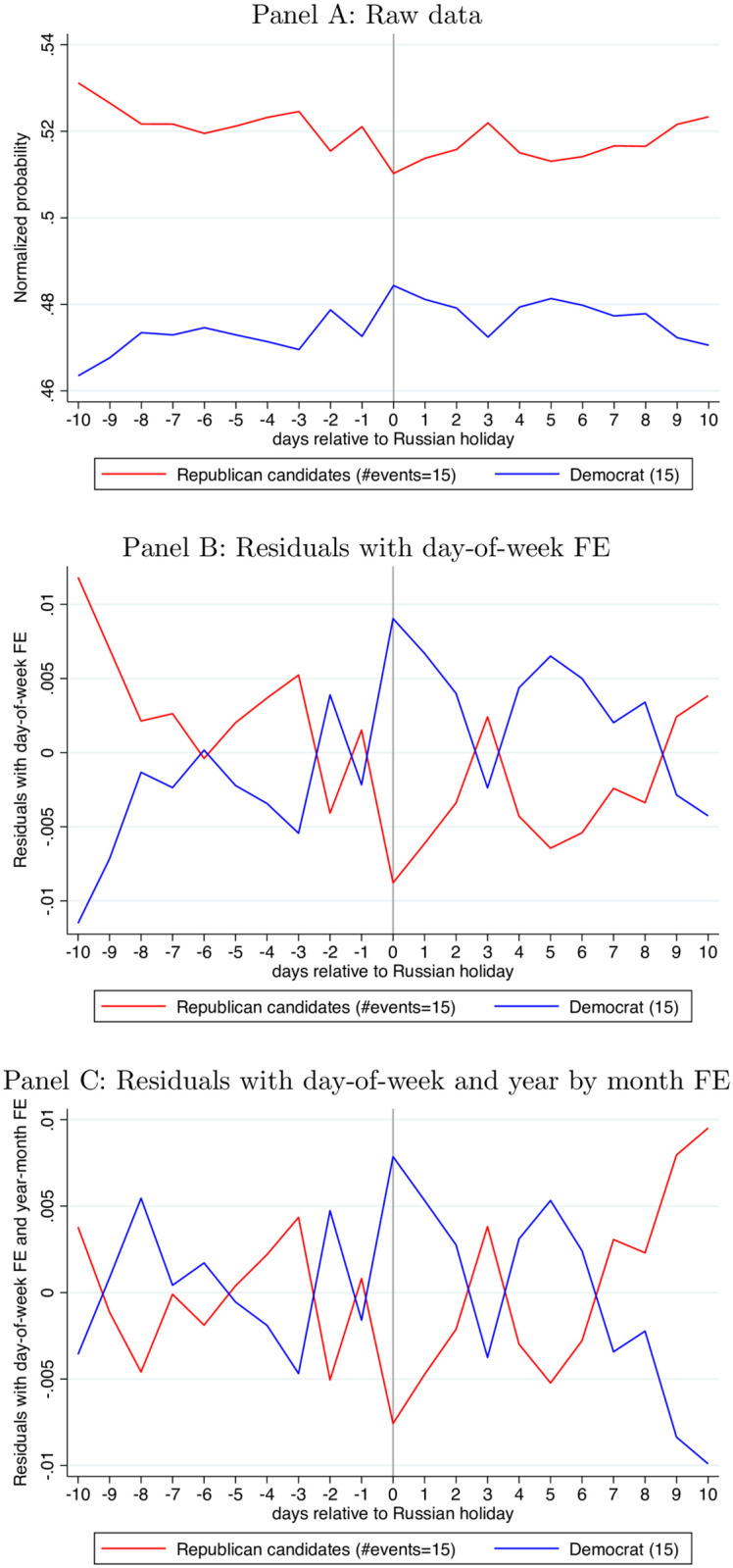
Event study of PredictIt betting odds around Russian holidays Nov 2014–Nov 2016. (There are 16 Russian holidays Nov 2014-Nov 2016. Similar to Fig 1, we only keep holiday events with complete data over the 21-day window. The last holiday, Nov 4, 2016, is dropped due to incomplete betting data). Panel A: Raw data. Panel B: Residuals with day-of-week FE. Panel C: Residuals with day-of-week and year by month FE.

We consider whether downstream outcomes—daily Hedonometer and in particular election odds—show a similar holiday pattern as IRA tweets. Fig 3 and Table 2 present our main results for election odds (see also S2.2 and S3.2 sections in S1 File). The most striking finding is that Republican odds for the 2016 Presidential Election hit their nadir on Russian holidays and Democratic odds hit their peak (Fig 3).

**Table 2:** Reduced form: Event study of PredictIt 2016 election odds around Russian holidays Nov. 2014–Nov. 2016.

	Panel A: Republican’s probability					
	(1)	(2)	(3)	(4)	(5)	(6)
Holiday_RU	-0.009**	-0.009***	-0.009**	-0.009**	-0.009***	-0.009***
	(0.004)	(0.003)	(0.003)	(0.003)	(0.003)	(0.003)
Holiday_US	-0.003	-0.003	-0.002	-0.002	-0.004	0.000
	(0.004)	(0.004)	(0.004)	(0.004)	(0.003)	(0.003)
Days (×10^−2^)		0.015	0.047	-0.001		0.291***
		(0.046)	(0.058)	(0.126)		(0.103)
Days^2^ (×10^−4^)			-0.004	0.018		-0.046
			(0.006)	(0.058)		(0.031)
Days^3^ (×10^−6^)				-.00221		.00092
				(.00641)		(.00283)
Observations	315	315	315	315	315	315
R-square	0.935	0.935	0.943	0.945	0.960	0.966
Y-mean	0.519	0.519	0.519	0.519	0.519	0.519
Y-std.dev.	0.081	0.081	0.081	0.081	0.081	0.081
Replications	1000	1000	1000	1000	1000	1000
	Panel B: Democrat’s probability					
Holiday_RU	0.010**	0.009***	0.009***	0.009***	0.010***	0.009***
	(0.004)	(0.003)	(0.003)	(0.003)	(0.003)	(0.003)
Holiday_US	0.003	0.003	0.002	0.002	0.004	-0.000
	(0.004)	(0.004)	(0.004)	(0.004)	(0.003)	(0.003)
Days (×10^−2^)		-0.021	-0.042	-0.036		-0.305***
		(0.046)	(0.058)	(0.120)		(0.103)
Days^2^ (×10^−4^)			0.003	-0.000		0.054*
			(0.006)	(0.054)		(0.032)
Days^3^ (×10^−6^)				.000285		-.00196
				(.00606)		(.00298)
Observations	315	315	315	315	315	315
R-square	0.936	0.936	0.940	0.940	0.953	0.960
Y-mean	0.475	0.475	0.475	0.475	0.475	0.475
Y-std.dev.	0.075	0.075	0.075	0.075	0.075	0.075
Replications	1000	1000	1000	1000	1000	1000
DOW FEs	Y	Y	Y	Y	Y	Y
Month FEs	Y	Y	Y	Y		
Year FEs	Y	Y	Y	Y		
Year-Month FEs					Y	Y

*Notes*: We cluster standard errors at the event level since the sampling is clustered, #clusters = 15. Given the bias that may result from having few clusters [15], we draw samples of the same size as the data (n = 315) and perform 1000 replications. Distinct event identifiers are generated in each bootstrap resample. Bootstrap standard errors are clustered at the distinct event level, #clusters = 15000.

* significant 10% level;

** significant at 5% level;

*** significant at 1% level.

### Hedonometer

While not our primary focus, analyzing the Hedonometer has the advantage of leveraging daily variation across the full time period of wave 1 tweets. The Hedonometer measures sentiment and inferred happiness of English-language tweets. Reduced form impacts for Russian holidays are not distinguishable from 0, implying no detectable impact on happiness. Standard errors are similar to point estimates. If we take double the point estimates, we can reject impacts of around.01. For comparison, we see that the Hedonometer increases.056 on US holidays, fell 0.06 when Brett Kavanaugh was confirmed for the Supreme Court, and fell.2 on the day of the mass shooting in Dayton (S20 Table in S1 File).

In S2.3 Section in S1 File, we evaluate stock market responses to Russian holidays, motivated by existing work documenting co-movements between online sentiment and stock-related outcomes, including market volatility [16, 17], stock or Bitcoin returns [18, 19], and trading volumes [20, 21]. Results in S13 Table in S1 File show neither Russian holidays nor US holidays affect financial indices. As Hedonometer also does not respond to Russian holidays, our null finding is consistent with a more targeted effect of Russian trolling and the existing work on social media sentiment and financial markets.

### 2016 & 2020 election odds

Daily presidential election odds as inferred from betting markets can reflect whether Russian interference was successful. A drawback in considering election odds is that we are forced to analyze a shorter time period, which renders our reduced form (but not the first stage) noisier.

Considering the 2016 election odds, **Iowa Election Markets data** began November 17, 2014 and ended November 10, 2016. This means we can analyze 2016 odds around 15 holiday events(Nov 4, 2016, is dropped due to incomplete “post” betting data) and 315 betting days in total. Using a 21 day window around each holiday, which prevents overlap between Fatherland and Women’s Day holidays. (If we drop the busiest trolling days and events, we analyze only 11 holidays and 231 days for all and original tweets, and 15 holidays and 315 days for retweeted tweets).

Democratic odds peak on Russian holidays, while Republican odds approach their nadir. The reduced form event study figures are shown in S2 Fig in S1 File: unadjusted event study estimates (top panel) are very similar to the regression-adjusted ones (middle and bottom panels). S17 and S18 Tables in S1 File show a statistically significant increase in 2016 Democratic odds on Russian holidays when trolling fell by around a third (Fig 2). S3.1 Section in S1 File shows first stage in tables for the November 2014-November 2016 period. If anything, the holiday-indued reduction is more obvious than in the full sample.

Conversely, odds for Republicans fell by a similar amount on the 15 Russian holidays. Notably, the reduced form estimates do not change *at all* with alternative sets of control variable in S17 and S18 Tables in S1 File—including month by year fixed effects—suggesting a robust relationship.

Fig 3 and Table 2 repeat the analysis of 2016 election odds using data from the **PredictIt betting market**. We sum prices from the candidate-specific market as it permits analysis of more holidays and more betting days (315 betting days, same as with the the Iowa data). The candidate-specific PredictIt market is substantially thicker than the political party PredictIt market, the former having nine times more trading volume over our sample period.

While newer, the PredictIt market is much thicker than the Iowa Election Market. Indeed, the trading volume over our 315 day period is two orders of magnitude higher on PredictIt than Iowa Election Markets (we thank Koleman Strumpf for flagging the differences in thickness/liquidity across exchanges). Additionally, only US Citizens may bet on the PredictIt market for presidential candidates, appealing for a reason we note below. (PredictIt’s Parker Howell emailed us on October 19, 2020: “All traders on PredictIt have to be American citizens who have passed ID verification. Anyone trying to trade from a Russian IP address would have been blocked in 2016. If ever we suspect a trader of fraud their account is frozen until they can upload a US-issued photo ID.”).

Fig 3 shows that the unadjusted reduced form for 2016 again shows a Democratic peak in odds on Russian holidays and a Republican minimum. And again, this basic pattern is unaffected by regression adjustment. (The vertical scale makes the regression-adjusted pattern appear noisier than the unadjusted reduced form Fig 3). Table 2 shows the reduced form effect of holidays is robust and distinguishable from 0 at the 1% significance level (5% significance level for Column 1 only). We block bootstrap standard errors at the event level to account for correlation within the small number of groups. Effect magnitudes are around.01. If in instrumental-variables fashion, one is willing to scale this by the first stage to extrapolate to the elimination of Russian trolling (see Section ‘Interpretation’), 2016 odds move by around.03. In contrast, US holidays show no impact on election odds (Table 2). Finally, we note Russian holidays have similar-sized effects in the Iowa and PredictIt data. The Democrats’ probability increases by 2.5% on Russian holidays in the Iowa data (S17 Table in S1 File) and increases 2.1% in the PredictIt data (Table 2). Conversely, Republicans’ probability decreases by 2.8% on Russian holidays in the Iowa data (S17 Table in S1 File), and decreases by 1.7% in the PredictIt data (Table 2).

Our 2020 election odds data begin in November 2016. Wave 1 tweets stop at the end of 2017. This permits a reduced form analysis of just 168 betting days (and 8 holiday events in total) that are, moreover, *extremely early* in the 2020 campaign. While holidays if anything matter more for the first stage in original tweets from late 2016 through 2017, in the reduced form we detect no impact on 2020 election odds that is distinguishable statistically from zero.

### Interpretation

The above reduced form estimates for holidays can be interpreted causally if the conditional independence assumption alone is satisfied [15]. *A priori*, we have no reason to think idiosyncratic Russian holidays should affect US presidential election odds absent Russian interference. Moreover, there is a plausible mechanism by which Russian holidays could affect election odds via interference: we show empirically trolling falls and trolling was specifically intended to help Trump’s campaign, according to the Senate Intelligence Committee 2019 (bipartisan) report. Thus, there is less pro-Trump interference on Russian holidays.

Non-interference channels by which Russian holidays could affect election odds we view as substantially less plausible. For example, non-trolling Russians living in the US might celebrate Russian holidays, impacting their leisure activities or cheerfulness and in turn their betting on the US election (or the Hedonometer). But as only US citizens can bet on the PredictIt election market, we are less concerned about direct effects of Russian citizens (in the US or in Russia). US citizens who observe Russian holidays and unconsciously affect election odds on Russian holidays strikes us as implausible. That US holidays appear to have no effect on US election odds (*cf*. the Hedonometer increase on US holidays) may suggest the *direct* effect of Russian holidays on betting behavior by Russian-Americans in PredictIt markets might also be modest.

Thus, we think Russian holidays impact US election odds when deliberate Russian election interference clearly falls. Still, we choose not to conduct an instrumental variables (IV) analysis because we lack a *comprehensive* measure of trolling specifically or Russian election interference more broadly, i.e. the endogenous variable. [15] note:

*The exclusion restriction is distinct from the claim that the instrument is (as good as) randomly assigned. Rather, it is a claim about a unique channel for causal effects of the instrument*.

This matters because Twitter was not the only online platform the IRA has targeted. Part of the holiday effect could be coming from variation in trolling on platforms like Facebook or Instagram, which would not be captured by the endogenous variable Twitter provided us. Additionally, Russian Twitter accounts that were not blocked could be undetected Trolls and follow a similar (unobserved) first stage on Twitter. Either of these channels would violate the exclusion restriction for the blocked suspected-IRA tweets (endogenous variable) we can observe and analyze. These plausible scenarios undermine the exclusion restriction, and therefore our ability to interpret IV estimates causally. (If we ignore these concerns and conduct an IV analysis, instrumented trolling—using holidays as the *Z*—relates to election odds in the expected way.) But importantly, they do not threaten our causal interpretation of the reduced form.

Our avoidance of IV even after demonstrating a relevant first stage and reduced form is not unique in applied microeconomics. For example, [22, 23] express concerns about their exclusion restrictions and emphasize instead a causal interpretation of the reduced form, as does [24] in a randomized control trial (RCT) design. Here, IV would incorrectly ascribe all holiday-induced changes in election odds to the IRA trolls that Twitter caught.

## Discussion

International political interference is anything but new. Britain interfered in the 1940 U.S. election to support Roosevelt. The CIA covertly placed ideological content in East German media [25] and orchestrated the overthrow of Iran’s democratically-elected prime minister Mohammad Mossadeqin in 1953. The CIA also interfered in the 1996 Russian election. Scott Shane (*The New York Times*) noted in a 2018 interview:

*But we have certainly put our thumb on the scales in elections once in Russia, trying to prevent the election of a communist in 1996 who was leading in the polls against Boris Yeltsin. And so we, you know, we intervened in a significant way to help Yeltsin’s re-election*.

What is more novel about election interference by the IRA is that it “industrialized the art of trolling” [1]. Indeed, a former IRA employee relayed to the Washington Post: “Your first feeling, when you ended up there, was that you were in some kind of factory that turned lying, telling untruths, into an industrial assembly line….”

The null findings in [4] resonate with a broader suspicion that while salacious, Russian trolling does not have important “downstream” effects, say to election outcomes. As the *New Yorker* recently put it, the impactful IRA narrative may be an “overly convenient” explanation for our home-grown problems and:

*What if, to borrow an old horror-movie trope, the call is coming from inside the house?* [25]

That is, domestic election interference by the President, news media disinformation, domestic conspiracy theories, etc. may be more consequential. President Trump himself has repeatedly questioned the integrity of mail-in voting and the Director of the US Postal Service has been accused of deliberately slowing mail deliveries. Switching to COVID-19, [26] attribute higher mortality to watching Sean Hannity on Fox, who has consistently downplayed the risks of infection. These culprits are all domestic.

Our novel approach to trolling attempts to isolate variation in disinformation coming from abroad. We find that Russian holidays and temperatures in St. Petersburg help predict daily variation in Russian trolling activity. That is, we argue a phone call is indeed also coming from “outside the house”.

Moreover, if Russian holidays and temperature are exogenous, then they may help address the issue for causal inference posed by endogenous selection into who accesses [4] or is the target of [5] IRA content. And if additional exogenous factors can be identified, this could be a powerful alternative approach for assessing trolling impacts. As troll tweets include a time stamp, this might include utilizing variation at a higher frequency than the daily variation we consider. For example, election odds are available from BetData by the hour. When Twitter discloses more recent Troll activity, these again can be explored for their relationship to exogenous drivers and thereby their potential causal effect on recent election-related outcomes. Twitter accounts from Turkey, China, Iran, and Venezuela have also been blocked by Twitter alongside those from Russia, so our approach could be extended to consider other source country-specific factors behind trolling.

Consistent with the Senate Intelligence Committee’s 2019 (bipartisan) report that Russian trolling was attempting to help the Trump campaign, our estimates for 2016 election odds suggest this effort may have achieved some success. Our reduced form approach readily generalizes to the analysis of additional high-frequency outcomes that may be of interest to researchers and are believed to impact elections, such as time-series variation in political campaign donations, US street protests, etc. Our analysis here can be viewed as initial “proof of concept” for future analyses that emphasize:

1. Exogenous drivers of Russian trolling activity—neglected by the existing literature.
2. Trolling’s causal effect on indirectly-affected outcomes of interest, i.e. outcomes where the precise path of content sharing, dissemination, and downstream impacts cannot be traced via social media platforms.

These indirect channels may be the most challenging and important ones to understand.

## Supporting information

S1 File (PDF)
