# Supplementary material for: Reduced trolling on Russian holidays and daily US Presidential election odds
Source: PLoS One. 2022 Mar 30;17(3):e0264507. doi: 10.1371/journal.pone.0264507 (PMC8966999; doi:10.1371/journal.pone.0264507)
Supplement: S1 File — (PDF) [file pone.0264507.s001.pdf]

Supporting Information: Reduced Trolling on Russian Holidays and Daily US Presidential Election Odds

Douglas Almond[*], Xinming Du[†], and Alana Vogel[‡]

February 16, 2022

Contents

S1 Robustness of Table 1 First Stage **2**
- S1.1 Dropping temperature variable 2
- S1.2 Dropping top 10 busiest tweeting days 4
- S1.3 Adding other major cities' temperature 7
- S1.4 Shortening time period toward Wave 1 activity 10
- S1.5 Adding other weather variables 16
- S1.6 Holiday week January 1-7 19

S2 Holiday Event Study (Regression Tables) **21**
- S2.1 First stage 21
- S2.2 Reduced form for Hedonometer and 2020 election odds 27
- S2.3 Reduced form for financial indexes 29
- S2.4 Robustness of the first stage 30

S3 Holiday Event Study Nov 2014-Nov 2016 **32**
- S3.1 First stage 32
- S3.2 Reduced form for Iowa 2016 election odds 35

S4 Regression Discontinuity Estimates of First Stage **38**

S5 Hedonometer's Response to US Events **40**

S6 Robustness with Blank Tweets **41**

S7 Examples of Twitter-identified Troll Tweets **47**

[*]Columbia University and NBER: da2152@columbia.edu
[†]Columbia University: xd2197@columbia.edu
[‡]Columbia University: asv2130@columbia.edu

Figure S1: Time series of tweet activity by wave of public release.

(We drop top 10 busiest days for wave 1, 2, 4 2012-2019 and calculate monthly sum of all tweets.)

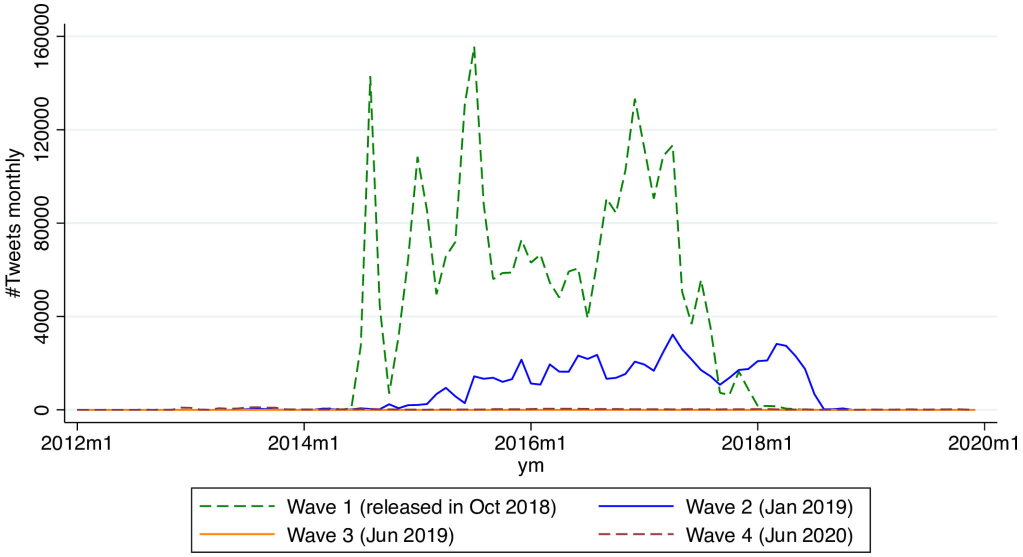

S1 Robustness of Table 1 First Stage

S1.1 Dropping temperature variable

Table S1: First stage: Russian holiday on blocked tweets on the day shift

	Panel A: #All tweets (z)					
	(1)	(2)	(3)	(4)	(5)	(6)
Holiday_RU × Wave=1	-0.340***	-0.339***	-0.339***	-0.339***	-0.340***	-0.340***
	(0.103)	(0.103)	(0.103)	(0.103)	(0.092)	(0.092)
Holiday_RU × Wave=2	-0.039	-0.038	-0.038	-0.038	-0.039	-0.039
	(0.103)	(0.103)	(0.103)	(0.103)	(0.092)	(0.092)
Holiday_RU × Wave=3	-0.005	-0.004	-0.004	-0.004	-0.005	-0.005
	(0.103)	(0.103)	(0.103)	(0.103)	(0.092)	(0.092)
Holiday_RU × Wave=4	-0.007	-0.006	-0.006	-0.006	-0.007	-0.007
	(0.103)	(0.103)	(0.103)	(0.103)	(0.092)	(0.092)
Holiday_US	.0134	.0135	.0136	.0136	.0139	.014
	(.047)	(.047)	(.0469)	(.0469)	(.0422)	(.0422)
Days ($\times 10^{-3}$)		.0803	.615	.837		-.0815
		(.849)	(.857)	(.871)		(2.27)
Days^2 ($\times 10^{-6}$)			-.187***	-.418**		.407
			(.0419)	(.171)		(1.79)
Days^3 ($\times 10^{-9}$)				.0527		-.128
				(.0379)		(.395)
Observations	11688	11688	11688	11688	11688	11688
R-square	0.365	0.365	0.366	0.366	0.502	0.502
Y-mean	167.8	167.8	167.8	167.8	167.8	167.8
Y-std.dev.	687.0	687.0	687.0	687.0	687.0	687.0
Y-mean Wave1	572.2	572.2	572.2	572.2	572.2	572.2
Y-std.dev. Wave1	1280.2	1280.2	1280.2	1280.2	1280.2	1280.2
Y-mean Wave2	93.36	93.36	93.36	93.36	93.36	93.36
Y-std.dev. Wave2	159.7	159.7	159.7	159.7	159.7	159.7
Y-mean Wave3	0.0010	0.0010	0.0010	0.0010	0.0010	0.0010
Y-std.dev. Wave3	0.0414	0.0414	0.0414	0.0414	0.0414	0.0414
Y-mean Wave4	5.785	5.785	5.785	5.785	5.785	5.785
Y-std.dev. Wave4	19.28	19.28	19.28	19.28	19.28	19.28
	Panel B: #Original tweets (z)					
Holiday_RU × Wave=1	-0.316***	-0.315***	-0.315***	-0.315***	-0.315***	-0.315***
	(0.109)	(0.109)	(0.109)	(0.109)	(0.100)	(0.100)
Holiday_RU × Wave=2	-0.023	-0.023	-0.023	-0.023	-0.024	-0.023
	(0.109)	(0.109)	(0.109)	(0.109)	(0.100)	(0.100)
Holiday_RU × Wave=3	-0.006	-0.005	-0.005	-0.005	-0.006	-0.005
	(0.109)	(0.109)	(0.109)	(0.109)	(0.100)	(0.100)
Holiday_RU × Wave=4	-0.008	-0.007	-0.007	-0.007	-0.008	-0.007

	(0.109)	(0.109)	(0.109)	(0.109)	(0.100)	(0.100)
Holiday_US	.0298	.0299	.0299	.03	.0304	.0305
	(.0498)	(.0498)	(.0498)	(.0497)	(.0456)	(.0456)
Days ($\times 10^{-3}$)		.0994	.44	1.18		.0431
		(.9)	(.908)	(.923)		(2.45)
Days^2 ($\times 10^{-6}$)			-.119***	-.887***		.254
			(.0444)	(.181)		(1.94)
Days^3 ($\times 10^{-9}$)				.175***		-.0834
				(.0401)		(.428)
Observations	11688	11688	11688	11688	11688	11688
R-square	0.287	0.287	0.287	0.289	0.418	0.418
Y-mean	100.4	100.4	100.4	100.4	100.4	100.4
Y-std.dev.	564.0	564.0	564.0	564.0	564.0	564.0
Y-mean Wave1	375.4	375.4	375.4	375.4	375.4	375.4
Y-std.dev. Wave1	1080.7	1080.7	1080.7	1080.7	1080.7	1080.7
Y-mean Wave2	21.65	21.65	21.65	21.65	21.65	21.65
Y-std.dev. Wave2	58.68	58.68	58.68	58.68	58.68	58.68
Y-mean Wave3	0.0010	0.0010	0.0010	0.0010	0.0010	0.0010
Y-std.dev. Wave3	0.0414	0.0414	0.0414	0.0414	0.0414	0.0414
Y-mean Wave4	4.430	4.430	4.430	4.430	4.430	4.430
Y-std.dev. Wave4	18.82	18.82	18.82	18.82	18.82	18.82
	Panel C: #Retweeted tweets (z)					
Holiday_RU × Wave=1	-0.195*	-0.195*	-0.195*	-0.195*	-0.195**	-0.196**
	(0.102)	(0.102)	(0.102)	(0.102)	(0.084)	(0.084)
Holiday_RU × Wave=2	-0.048	-0.048	-0.048	-0.048	-0.048	-0.049
	(0.102)	(0.102)	(0.102)	(0.102)	(0.084)	(0.084)
Holiday_RU × Wave=3	-0.000	-0.000	-0.000	-0.000	-0.000	-0.001
	(0.102)	(0.102)	(0.102)	(0.102)	(0.084)	(0.084)
Holiday_RU × Wave=4	-0.002	-0.002	-0.002	-0.002	-0.002	-0.002
	(0.102)	(0.102)	(0.102)	(0.102)	(0.084)	(0.084)
Holiday_US	-.0267	-.0267	-.0266	-.0267	-.0268	-.0268
	(.0467)	(.0467)	(.0466)	(.0466)	(.0385)	(.0385)
Days ($\times 10^{-3}$)		-.00302	.614	-.317		-.282
		(.844)	(.851)	(.865)		(2.07)
Days^2 ($\times 10^{-6}$)			-.216***	.75***		.481
			(.0416)	(.17)		(1.64)
Days^3 ($\times 10^{-9}$)				-.221***		-.144
				(.0376)		(.362)
Observations	11688	11688	11688	11688	11688	11688
R-square	0.372	0.372	0.374	0.376	0.584	0.584
Y-mean	67.46	67.46	67.46	67.46	67.46	67.46
Y-std.dev.	284.1	284.1	284.1	284.1	284.1	284.1
Y-mean Wave1	196.8	196.8	196.8	196.8	196.8	196.8
Y-std.dev. Wave1	530.1	530.1	530.1	530.1	530.1	530.1

Y-mean Wave2	71.70	71.70	71.70	71.70	71.70	71.70
Y-std.dev. Wave2	127.6	127.6	127.6	127.6	127.6	127.6
Y-mean Wave3	0	0	0	0	0	0
Y-std.dev. Wave3	0	0	0	0	0	0
Y-mean Wave4	1.355	1.355	1.355	1.355	1.355	1.355
Y-std.dev. Wave4	2.660	2.660	2.660	2.660	2.660	2.660
DOW FEs	Y	Y	Y	Y	Y	Y
Wave-Month FEs	Y	Y	Y	Y		
Wave-Year FEs	Y	Y	Y	Y		
Wave-Year-Month FEs					Y	Y

Notes: * significant 10% level; ** significant at 5% level; *** significant at 1% level.

S1.2 Dropping top 10 busiest tweeting days

Table S2: First stage: Russian holiday and temperature on blocked tweets on the day shift

	Panel A: #All tweets (z)					
	(1)	(2)	(3)	(4)	(5)	(6)
Holiday_RU × Wave=1	-0.302***	-0.297***	-0.297***	-0.297***	-0.305***	-0.302***
	(0.093)	(0.093)	(0.093)	(0.093)	(0.077)	(0.077)
Holiday_RU × Wave=2	-0.047	-0.042	-0.042	-0.042	-0.046	-0.043
	(0.093)	(0.093)	(0.093)	(0.093)	(0.077)	(0.077)
Holiday_RU × Wave=3	-0.006	-0.001	-0.001	-0.001	-0.006	-0.002
	(0.093)	(0.093)	(0.093)	(0.093)	(0.077)	(0.077)
Holiday_RU × Wave=4	-0.007	-0.002	-0.003	-0.003	-0.006	-0.003
	(0.094)	(0.094)	(0.094)	(0.094)	(0.077)	(0.077)
Temperature × Wave=1 (z)	-.0456	-.0457	-.0469	-.0465	-.067**	-.0672**
	(.0292)	(.0292)	(.0291)	(.0291)	(.0275)	(.0275)
Temperature × Wave=2 (z)	-.0178	-.0178	-.0191	-.0187	.00316	.00301
	(.0292)	(.0292)	(.0291)	(.0291)	(.0275)	(.0275)
Temperature × Wave=3 (z)	.000916	.000897	-.000344	.0000836	.00144	.00129
	(.0291)	(.0291)	(.0291)	(.0291)	(.0275)	(.0275)
Temperature × Wave=4 (z)	.000177	.000165	-.00138	-.000938	.00128	.00113
	(.0293)	(.0293)	(.0292)	(.0292)	(.0276)	(.0276)
Holiday_US	.031	.0315	.0313	.0312	.0291	.0295
	(.0428)	(.0428)	(.0427)	(.0427)	(.0353)	(.0353)
Days ($\times 10^{-3}$)		.578	1.29*	1.19		-.233
		(.77)	(.776)	(.79)		(1.89)
Days^2 ($\times 10^{-6}$)			-.25***	-.154		1.05
			(.038)	(.155)		(1.49)
Days^3 ($\times 10^{-9}$)				-.0218		-.284

				(.0344)		(.33)
Observations	11531	11531	11531	11531	11531	11531
R-square	0.479	0.479	0.481	0.481	0.655	0.655
Y-mean	154.6	154.6	154.6	154.6	154.6	154.6
Y-std.dev.	544.0	544.0	544.0	544.0	544.0	544.0
Y-mean Wave1	523.9	523.9	523.9	523.9	523.9	523.9
Y-std.dev. Wave1	988.4	988.4	988.4	988.4	988.4	988.4
Y-mean Wave2	89.88	89.88	89.88	89.88	89.88	89.88
Y-std.dev. Wave2	145.7	145.7	145.7	145.7	145.7	145.7
Y-mean Wave3	0.0010	0.0010	0.0010	0.0010	0.0010	0.0010
Y-std.dev. Wave3	0.0416	0.0416	0.0416	0.0416	0.0416	0.0416
Y-mean Wave4	5.212	5.212	5.212	5.212	5.212	5.212
Y-std.dev. Wave4	5.824	5.824	5.824	5.824	5.824	5.824
	Panel B: #Original tweets (z)					
Holiday_RU × Wave=1	-0.268***	-0.262***	-0.262***	-0.262***	-0.278***	-0.273***
	(0.096)	(0.096)	(0.096)	(0.096)	(0.080)	(0.081)
Holiday_RU × Wave=2	-0.027	-0.022	-0.021	-0.022	-0.027	-0.022
	(0.096)	(0.096)	(0.096)	(0.096)	(0.080)	(0.081)
Holiday_RU × Wave=3	-0.008	-0.002	-0.002	-0.002	-0.008	-0.003
	(0.096)	(0.096)	(0.096)	(0.096)	(0.080)	(0.081)
Holiday_RU × Wave=4	-0.009	-0.003	-0.004	-0.004	-0.009	-0.004
	(0.097)	(0.097)	(0.097)	(0.097)	(0.081)	(0.081)
Temperature × Wave=1 (z)	-.0325	-.0325	-.0336	-.0355	-.0479*	-.048*
	(.0302)	(.0302)	(.0301)	(.0301)	(.0289)	(.0289)
Temperature × Wave=2 (z)	-.00225	-.00227	-.00332	-.00525	.00241	.00226
	(.0302)	(.0302)	(.0301)	(.0301)	(.0289)	(.0289)
Temperature × Wave=3 (z)	.00021	.000185	-.000812	-.00288	.000608	.000477
	(.0301)	(.0301)	(.0301)	(.0301)	(.0288)	(.0288)
Temperature × Wave=4 (z)	-.000359	-.000382	-.00167	-.00384	.000832	.000691
	(.0303)	(.0303)	(.0302)	(.0302)	(.029)	(.029)
Holiday_US	.0597	.0604	.0602	.0605	.0577	.0583
	(.0443)	(.0443)	(.0443)	(.0443)	(.0371)	(.0371)
Days ($\times 10^{-3}$)		.742	1.31	1.76**		.189
		(.797)	(.804)	(.818)		(1.98)
Days^2 ($\times 10^{-6}$)			-.201***	-.663***		.895
			(.0393)	(.161)		(1.57)
Days^3 ($\times 10^{-9}$)				.106***		-.258
				(.0356)		(.346)
Observations	11531	11531	11531	11531	11531	11531
R-square	0.435	0.436	0.437	0.437	0.615	0.615
Y-mean	87.08	87.08	87.08	87.08	87.08	87.08
Y-std.dev.	377.6	377.6	377.6	377.6	377.6	377.6
Y-mean Wave1	325.8	325.8	325.8	325.8	325.8	325.8
Y-std.dev. Wave1	702.6	702.6	702.6	702.6	702.6	702.6

Y-mean Wave2	18.91	18.91	18.91	18.91	18.91	18.91
Y-std.dev. Wave2	35.27	35.27	35.27	35.27	35.27	35.27
Y-mean Wave3	0.0010	0.0010	0.0010	0.0010	0.0010	0.0010
Y-std.dev. Wave3	0.0416	0.0416	0.0416	0.0416	0.0416	0.0416
Y-mean Wave4	3.908	3.908	3.908	3.908	3.908	3.908
Y-std.dev. Wave4	4.971	4.971	4.971	4.971	4.971	4.971
	Panel C: #Retweeted tweets (z)					
Holiday_RU × Wave=1	-0.206**	-0.206**	-0.205**	-0.205**	-0.209***	-0.210***
	(0.100)	(0.100)	(0.100)	(0.100)	(0.080)	(0.080)
Holiday_RU × Wave=2	-0.051	-0.051	-0.051	-0.051	-0.051	-0.052
	(0.100)	(0.100)	(0.100)	(0.100)	(0.080)	(0.080)
Holiday_RU × Wave=3	-0.000	-0.000	-0.000	-0.000	-0.000	-0.001
	(0.100)	(0.100)	(0.100)	(0.100)	(0.080)	(0.080)
Holiday_RU × Wave=4	-0.002	-0.002	-0.002	-0.002	-0.002	-0.003
	(0.100)	(0.100)	(0.100)	(0.100)	(0.080)	(0.080)
Temperature × Wave=1 (z)	-.0542*	-.0542*	-.0555*	-.0524*	-.0765***	-.0765***
	(.0314)	(.0314)	(.0313)	(.0313)	(.0286)	(.0286)
Temperature × Wave=2 (z)	-.0448	-.0448	-.0462	-.043	-.00413	-.00422
	(.0314)	(.0314)	(.0314)	(.0313)	(.0286)	(.0286)
Temperature × Wave=3 (z)	.00158	.00158	.000254	.00338	.00216	.00207
	(.0314)	(.0314)	(.0313)	(.0313)	(.0286)	(.0286)
Temperature × Wave=4 (z)	.00103	.00103	-.000707	.00264	.00188	.0018
	(.0315)	(.0315)	(.0315)	(.0315)	(.0287)	(.0287)
Holiday_US	-.0243	-.0243	-.0245	-.0251	-.0268	-.0269
	(.0462)	(.0462)	(.0462)	(.0461)	(.0369)	(.0369)
Days ($\times 10^{-3}$)		.00615	.762	.0839		-.304
		(.831)	(.837)	(.851)		(1.97)
Days^2 ($\times 10^{-6}$)			-.268***	.431**		.511
			(.0409)	(.167)		(1.55)
Days^3 ($\times 10^{-9}$)				-.16***		-.156
				(.0371)		(.344)
Observations	11530	11530	11530	11530	11530	11530
R-square	0.403	0.403	0.405	0.406	0.631	0.631
Y-mean	63.15	63.15	63.15	63.15	63.15	63.15
Y-std.dev.	246.8	246.8	246.8	246.8	246.8	246.8
Y-mean Wave1	182.1	182.1	182.1	182.1	182.1	182.1
Y-std.dev. Wave1	456.2	456.2	456.2	456.2	456.2	456.2
Y-mean Wave2	69.44	69.44	69.44	69.44	69.44	69.44
Y-std.dev. Wave2	116.8	116.8	116.8	116.8	116.8	116.8
Y-mean Wave3	0	0	0	0	0	0
Y-std.dev. Wave3	0	0	0	0	0	0
Y-mean Wave4	1.270	1.270	1.270	1.270	1.270	1.270
Y-std.dev. Wave4	2.202	2.202	2.202	2.202	2.202	2.202
DOW FEs	Y	Y	Y	Y	Y	Y

Wave-Month FEs	Y	Y	Y	Y		
Wave-Year FEs	Y	Y	Y	Y		
Wave-Year-Month FEs					Y	Y

Notes: * significant 10% level; ** significant at 5% level; *** significant at 1% level.

S1.3 Adding other major cities' temperature

Table S3: First stage: Russian holiday and temperature on blocked tweets on the day shift

	Panel A: #All tweets (z)					
	(1)	(2)	(3)	(4)	(5)	(6)
Holiday_RU × Wave=1	-0.360***	-0.358***	-0.357***	-0.357***	-0.355***	-0.354***
	(0.107)	(0.107)	(0.107)	(0.107)	(0.096)	(0.097)
Holiday_RU × Wave=2	-0.046	-0.043	-0.043	-0.043	-0.040	-0.039
	(0.107)	(0.107)	(0.107)	(0.107)	(0.096)	(0.097)
Holiday_RU × Wave=3	-0.009	-0.007	-0.006	-0.006	-0.003	-0.003
	(0.107)	(0.107)	(0.107)	(0.107)	(0.096)	(0.097)
Holiday_RU × Wave=4	-0.011	-0.009	-0.008	-0.009	-0.006	-0.005
	(0.107)	(0.107)	(0.107)	(0.107)	(0.096)	(0.097)
Temperature × Wave=1 (z)	.0627*	.0627*	.0612*	.06*	-.000265	-.000229
	(.0334)	(.0334)	(.0333)	(.0333)	(.0342)	(.0342)
Temperature × Wave=2 (z)	-.0111	-.0111	-.0126	-.0137	.00445	.00449
	(.0334)	(.0334)	(.0333)	(.0333)	(.0342)	(.0342)
Temperature × Wave=3 (z)	.00448	.00451	.00295	.00184	.00126	.00129
	(.0334)	(.0334)	(.0333)	(.0333)	(.0342)	(.0342)
Temperature × Wave=4 (z)	.00259	.00262	.00106	-.0000459	-.000118	-.000082
	(.0334)	(.0334)	(.0333)	(.0333)	(.0342)	(.0342)
Holiday_US	.0368	.0375	.0379	.0378	.0429	.0432
	(.0519)	(.052)	(.0519)	(.0519)	(.0467)	(.0467)
Temp_LosAngeles (z)	-.00754	-.00751	-.00348	-.00428	.0192	.0192
	(.0127)	(.0127)	(.0127)	(.0127)	(.0125)	(.0125)
Temp_NewYork (z)	-.108***	-.108***	-.11***	-.108***	-.0103	-.0104
	(.0365)	(.0365)	(.0365)	(.0365)	(.0346)	(.0346)
Temp_WashingtonDC (z)	.0963***	.0967***	.0995***	.0996***	.0171	.0173
	(.0355)	(.0355)	(.0355)	(.0355)	(.0337)	(.0338)
Temp_London (z)	-.0225	-.0226	-.0236*	-.0222	-.0205	-.0204
	(.0138)	(.0138)	(.0138)	(.0139)	(.0135)	(.0135)
Days ($\times 10^{-3}$)		.311	.875	1.07		.4
		(.885)	(.893)	(.909)		(2.56)
Days^2 ($\times 10^{-6}$)			-.193***	-.389**		.00903
			(.0433)	(.179)		(1.96)

Days^3 ($\times 10^{-9}$)				.0448		-.0364
				(.0397)		(.425)
Observations	11140	11140	11140	11140	11140	11140
R-square	0.363	0.363	0.364	0.364	0.500	0.500
Y-mean	172.4	172.4	172.4	172.4	172.4	172.4
Y-std.dev.	699.2	699.2	699.2	699.2	699.2	699.2
Y-mean Wave1	587.6	587.6	587.6	587.6	587.6	587.6
Y-std.dev. Wave1	1301.6	1301.6	1301.6	1301.6	1301.6	1301.6
Y-mean Wave2	96.13	96.13	96.13	96.13	96.13	96.13
Y-std.dev. Wave2	160.7	160.7	160.7	160.7	160.7	160.7
Y-mean Wave3	0.0011	0.0011	0.0011	0.0011	0.0011	0.0011
Y-std.dev. Wave3	0.0424	0.0424	0.0424	0.0424	0.0424	0.0424
Y-mean Wave4	5.971	5.971	5.971	5.971	5.971	5.971
Y-std.dev. Wave4	19.72	19.72	19.72	19.72	19.72	19.72

	Panel B: #Original tweets (z)					
Holiday_RU × Wave=1	-0.331***	-0.329***	-0.328***	-0.329***	-0.330***	-0.329***
	(0.114)	(0.114)	(0.114)	(0.114)	(0.104)	(0.104)
Holiday_RU × Wave=2	-0.024	-0.022	-0.021	-0.022	-0.023	-0.022
	(0.114)	(0.114)	(0.114)	(0.114)	(0.104)	(0.104)
Holiday_RU × Wave=3	-0.005	-0.003	-0.002	-0.003	-0.004	-0.003
	(0.114)	(0.114)	(0.114)	(0.114)	(0.104)	(0.104)
Holiday_RU × Wave=4	-0.007	-0.005	-0.004	-0.005	-0.006	-0.005
	(0.114)	(0.114)	(0.114)	(0.114)	(0.104)	(0.104)
Temperature × Wave=1 (z)	.104***	.104***	.103***	.0987***	.0251	.0252
	(.0353)	(.0353)	(.0353)	(.0353)	(.037)	(.037)
Temperature × Wave=2 (z)	.00943	.00946	.00841	.00447	.00815	.00826
	(.0353)	(.0353)	(.0353)	(.0353)	(.037)	(.037)
Temperature × Wave=3 (z)	.00355	.00358	.00252	-.00141	-.000683	-.000578
	(.0353)	(.0353)	(.0353)	(.0353)	(.037)	(.037)
Temperature × Wave=4 (z)	.00132	.00135	.000292	-.00364	-.0024	-.0023
	(.0353)	(.0353)	(.0353)	(.0353)	(.037)	(.037)
Holiday_US	.0567	.0574	.0576	.0575	.0595	.0599
	(.055)	(.0551)	(.055)	(.055)	(.0504)	(.0505)
Temp_LosAngeles (z)	.0147	.0147	.0174	.0146	.0242*	.0243*
	(.0134)	(.0134)	(.0134)	(.0135)	(.0135)	(.0135)
Temp_NewYork (z)	-.0906**	-.0909**	-.0921**	-.0842**	-.0231	-.0233
	(.0387)	(.0387)	(.0387)	(.0387)	(.0374)	(.0374)
Temp_WashingtonDC (z)	.0596	.06	.0619*	.0623*	.0304	.0308
	(.0376)	(.0376)	(.0376)	(.0376)	(.0364)	(.0365)
Temp_London (z)	-.0301**	-.0301**	-.0308**	-.0259*	-.0119	-.0119
	(.0147)	(.0147)	(.0147)	(.0147)	(.0146)	(.0146)
Days ($\times 10^{-3}$)		.303	.685	1.36		.658
		(.937)	(.946)	(.963)		(2.77)
Days^2 ($\times 10^{-6}$)			-.131***	-.827***		-.233

			(.0459)	(.19)		(2.12)
Days^3 ($\times 10^{-9}$)				.159***		.0232
				(.042)		(.459)
Observations	11140	11140	11140	11140	11140	11140
R-square	0.285	0.285	0.285	0.286	0.416	0.416
Y-mean	102.5	102.5	102.5	102.5	102.5	102.5
Y-std.dev.	573.8	573.8	573.8	573.8	573.8	573.8
Y-mean Wave1	383.5	383.5	383.5	383.5	383.5	383.5
Y-std.dev. Wave1	1099.2	1099.2	1099.2	1099.2	1099.2	1099.2
Y-mean Wave2	21.76	21.76	21.76	21.76	21.76	21.76
Y-std.dev. Wave2	55.92	55.92	55.92	55.92	55.92	55.92
Y-mean Wave3	0.0011	0.0011	0.0011	0.0011	0.0011	0.0011
Y-std.dev. Wave3	0.0424	0.0424	0.0424	0.0424	0.0424	0.0424
Y-mean Wave4	4.575	4.575	4.575	4.575	4.575	4.575
Y-std.dev. Wave4	19.26	19.26	19.26	19.26	19.26	19.26
	Panel C: #Retweeted tweets (z)					
Holiday_RU × Wave=1	-0.213**	-0.212**	-0.212**	-0.211**	-0.204**	-0.204**
	(0.107)	(0.107)	(0.107)	(0.107)	(0.088)	(0.088)
Holiday_RU × Wave=2	-0.062	-0.061	-0.061	-0.060	-0.050	-0.050
	(0.107)	(0.107)	(0.107)	(0.107)	(0.088)	(0.088)
Holiday_RU × Wave=3	-0.012	-0.010	-0.010	-0.009	-0.000	-0.001
	(0.107)	(0.107)	(0.107)	(0.107)	(0.088)	(0.088)
Holiday_RU × Wave=4	-0.013	-0.012	-0.011	-0.010	-0.002	-0.002
	(0.107)	(0.107)	(0.107)	(0.107)	(0.088)	(0.088)
Temperature × Wave=1 (z)	-.0543	-.0543	-.056*	-.0508	-.0505	-.0507
	(.0331)	(.0331)	(.0331)	(.0331)	(.0313)	(.0313)
Temperature × Wave=2 (z)	-.0456	-.0456	-.0472	-.0421	-.00542	-.00554
	(.0331)	(.0331)	(.0331)	(.0331)	(.0313)	(.0313)
Temperature × Wave=3 (z)	.00379	.0038	.00213	.00726	.0044	.00428
	(.0331)	(.0331)	(.0331)	(.0331)	(.0313)	(.0313)
Temperature × Wave=4 (z)	.00365	.00366	.00199	.00712	.00448	.00436
	(.0331)	(.0331)	(.0331)	(.0331)	(.0313)	(.0313)
Holiday_US	-.0235	-.0232	-.0228	-.0226	-.0144	-.0146
	(.0516)	(.0516)	(.0516)	(.0515)	(.0427)	(.0427)
Temp_LosAngeles (z)	-.0473***	-.0473***	-.043***	-.0393***	-.00156	-.00182
	(.0126)	(.0126)	(.0126)	(.0126)	(.0114)	(.0115)
Temp_NewYork (z)	-.0811**	-.0813**	-.0831**	-.0935***	.0209	.0211
	(.0363)	(.0363)	(.0362)	(.0362)	(.0317)	(.0317)
Temp_WashingtonDC (z)	.114***	.115***	.118***	.117***	-.0189	-.0192
	(.0353)	(.0353)	(.0353)	(.0352)	(.0308)	(.0309)
Temp_London (z)	.00518	.00515	.0041	-.00223	-.026**	-.0258**
	(.0137)	(.0138)	(.0137)	(.0138)	(.0124)	(.0124)
Days ($\times 10^{-3}$)		.15	.755	-.13		-.339
		(.879)	(.887)	(.902)		(2.34)

Days^2 ($\times 10^{-6}$)			-.207***	.701***		.484
			(.043)	(.178)		(1.79)
Days^3 ($\times 10^{-9}$)				-.207***		-.134
				(.0394)		(.389)
Observations	11140	11140	11140	11140	11140	11140
R-square	0.374	0.374	0.375	0.377	0.583	0.583
Y-mean	69.95	69.95	69.95	69.95	69.95	69.95
Y-std.dev.	298.8	298.8	298.8	298.8	298.8	298.8
Y-mean Wave1	196.8	196.8	196.8	196.8	196.8	196.8
Y-std.dev. Wave1	530.1	530.1	530.1	530.1	530.1	530.1
Y-mean Wave2	74.37	74.37	74.37	74.37	74.37	74.37
Y-std.dev. Wave2	129.5	129.5	129.5	129.5	129.5	129.5
Y-mean Wave3	0	0	0	0	0	0
Y-std.dev. Wave3	0	0	0	0	0	0
Y-mean Wave4	1.396	1.396	1.396	1.396	1.396	1.396
Y-std.dev. Wave4	2.700	2.700	2.700	2.700	2.700	2.700
DOW FEs	Y	Y	Y	Y	Y	Y
Wave-Month FEs	Y	Y	Y	Y		
Wave-Year FEs	Y	Y	Y	Y		
Wave-Year-Month FEs					Y	Y

Notes: The sample size is smaller than that in Table 1 is due to missing temperature data in newly added cities. * significant 10% level; ** significant at 5% level; *** significant at 1% level.

S1.4 Shortening time period toward Wave 1 activity

Table S4: First stage 2012-2017

	Panel A: #All tweets (z)					
	(1)	(2)	(3)	(4)	(5)	(6)
Holiday_RU × Wave=1	-0.394***	-0.392***	-0.392***	-0.392***	-0.393***	-0.393***
	(0.119)	(0.120)	(0.119)	(0.119)	(0.107)	(0.108)
Holiday_RU × Wave=2	-0.033	-0.032	-0.031	-0.031	-0.033	-0.033
	(0.119)	(0.120)	(0.119)	(0.119)	(0.107)	(0.108)
Holiday_RU × Wave=3	-0.002	-0.001	-0.001	-0.001	-0.002	-0.002
	(0.119)	(0.120)	(0.119)	(0.119)	(0.107)	(0.108)
Holiday_RU × Wave=4	-0.002	-0.001	-0.001	-0.001	-0.003	-0.002
	(0.119)	(0.120)	(0.119)	(0.119)	(0.107)	(0.108)
Temperature × Wave=1 (z)	.0586	.0586	.0545	.06	.00088	.0014
	(.0372)	(.0372)	(.0371)	(.0371)	(.0384)	(.0384)
Temperature × Wave=2 (z)	-.0059	-.00594	-.00998	-.00451	.00332	.00384
	(.0372)	(.0372)	(.0371)	(.0371)	(.0384)	(.0384)
Temperature × Wave=3 (z)	.00081	.000767	-.00328	.0022	.00134	.00185

	(.0372)	(.0372)	(.0371)	(.0371)	(.0384)	(.0384)
Temperature × Wave=4 (z)	.000835	.000791	-.00325	.00222	.00173	.00224
	(.0372)	(.0372)	(.0371)	(.0371)	(.0384)	(.0384)
Holiday_US	.0184	.0185	.0194	.0189	.0203	.0202
	(.0546)	(.0546)	(.0545)	(.0545)	(.0492)	(.0492)
Days ($\times 10^{-3}$)		.162	.948	-.0137		-.722
		(.99)	(.999)	(1.02)		(2.65)
Days^2 ($\times 10^{-6}$)			-.361***	.965***		1.28
			(.0653)	(.273)		(2.8)
Days^3 ($\times 10^{-9}$)				-.403***		-.425
				(.0806)		(.824)
Observations	8684	8684	8684	8684	8684	8684
R-square	0.357	0.357	0.360	0.362	0.492	0.492
Y-mean	213.8	213.8	213.8	213.8	213.8	213.8
Y-std.dev.	785.9	785.9	785.9	785.9	785.9	785.9
Y-mean Wave1	756.7	756.7	756.7	756.7	756.7	756.7
Y-std.dev. Wave1	1431.5	1431.5	1431.5	1431.5	1431.5	1431.5
Y-mean Wave2	92.58	92.58	92.58	92.58	92.58	92.58
Y-std.dev. Wave2	154.0	154.0	154.0	154.0	154.0	154.0
Y-mean Wave3	0	0	0	0	0	0
Y-std.dev. Wave3	0	0	0	0	0	0
Y-mean Wave4	5.891	5.891	5.891	5.891	5.891	5.891
Y-std.dev. Wave4	8.448	8.448	8.448	8.448	8.448	8.448
	Panel B: #Original tweets (z)					
Holiday_RU × Wave=1	-0.366***	-0.365***	-0.365***	-0.365***	-0.365***	-0.364***
	(0.126)	(0.126)	(0.126)	(0.126)	(0.116)	(0.116)
Holiday_RU × Wave=2	-0.021	-0.020	-0.020	-0.020	-0.021	-0.021
	(0.126)	(0.126)	(0.126)	(0.126)	(0.116)	(0.116)
Holiday_RU × Wave=3	-0.004	-0.003	-0.003	-0.003	-0.005	-0.004
	(0.126)	(0.126)	(0.126)	(0.126)	(0.116)	(0.116)
Holiday_RU × Wave=4	-0.004	-0.003	-0.003	-0.003	-0.004	-0.004
	(0.126)	(0.126)	(0.126)	(0.126)	(0.116)	(0.116)
Temperature × Wave=1 (z)	.1**	.1**	.0962**	.0966**	.0339	.0342
	(.0393)	(.0393)	(.0392)	(.0392)	(.0413)	(.0414)
Temperature × Wave=2 (z)	.00727	.00724	.00327	.00363	.00966	.0099
	(.0393)	(.0393)	(.0392)	(.0392)	(.0413)	(.0414)
Temperature × Wave=3 (z)	.000272	.00024	-.00373	-.00336	.000638	.000878
	(.0393)	(.0393)	(.0392)	(.0392)	(.0413)	(.0414)
Temperature × Wave=4 (z)	.000132	.0000999	-.00387	-.0035	.000926	.00117
	(.0393)	(.0393)	(.0392)	(.0392)	(.0413)	(.0414)
Holiday_US	.0347	.0348	.0356	.0356	.0373	.0372
	(.0577)	(.0577)	(.0576)	(.0576)	(.0529)	(.053)
Days ($\times 10^{-3}$)		.118	.889	.825		-.213
		(1.05)	(1.05)	(1.07)		(2.85)

Days^2 ($\times 10^{-6}$)			-.354***	-.266		.513
			(.069)	(.289)		(3.01)
Days^3 ($\times 10^{-9}$)				-.0267		-.181
				(.0852)		(.887)
Observations	8684	8684	8684	8684	8684	8684
R-square	0.282	0.282	0.284	0.284	0.410	0.410
Y-mean	129.6	129.6	129.6	129.6	129.6	129.6
Y-std.dev.	647.2	647.2	647.2	647.2	647.2	647.2
Y-mean Wave1	493.0	493.0	493.0	493.0	493.0	493.0
Y-std.dev. Wave1	1223.2	1223.2	1223.2	1223.2	1223.2	1223.2
Y-mean Wave2	20.44	20.44	20.44	20.44	20.44	20.44
Y-std.dev. Wave2	58.15	58.15	58.15	58.15	58.15	58.15
Y-mean Wave3	0	0	0	0	0	0
Y-std.dev. Wave3	0	0	0	0	0	0
Y-mean Wave4	4.743	4.743	4.743	4.743	4.743	4.743
Y-std.dev. Wave4	7.074	7.074	7.074	7.074	7.074	7.074
	Panel C: #Retweeted tweets (z)					
Holiday_RU × Wave=1	-0.223*	-0.222*	-0.222*	-0.221*	-0.224**	-0.224**
	(0.119)	(0.119)	(0.119)	(0.118)	(0.099)	(0.099)
Holiday_RU × Wave=2	-0.039	-0.037	-0.037	-0.037	-0.039	-0.039
	(0.119)	(0.119)	(0.119)	(0.118)	(0.099)	(0.099)
Holiday_RU × Wave=3	0.004	0.005	0.005	0.005	0.004	0.004
	(0.119)	(0.119)	(0.119)	(0.118)	(0.099)	(0.099)
Holiday_RU × Wave=4	0.002	0.004	0.004	0.004	0.003	0.003
	(0.119)	(0.119)	(0.119)	(0.118)	(0.099)	(0.099)
Temperature × Wave=1 (z)	-.0584	-.0584	-.0603	-.0477	-.0658*	-.065*
	(.037)	(.037)	(.037)	(.0367)	(.0352)	(.0352)
Temperature × Wave=2 (z)	-.0289	-.0289	-.0308	-.0182	-.0113	-.0105
	(.037)	(.037)	(.037)	(.0367)	(.0352)	(.0352)
Temperature × Wave=3 (z)	.00142	.00138	-.000493	.0121	.00196	.00274
	(.037)	(.037)	(.037)	(.0367)	(.0352)	(.0352)
Temperature × Wave=4 (z)	.00176	.00172	-.000153	.0124	.00234	.00311
	(.037)	(.037)	(.037)	(.0367)	(.0352)	(.0352)
Holiday_US	-.0249	-.0248	-.0244	-.0255	-.0253	-.0255
	(.0544)	(.0544)	(.0543)	(.0539)	(.0451)	(.0451)
Days ($\times 10^{-3}$)		.156	.52	-1.69*		-1.33
		(.985)	(.995)	(1.01)		(2.43)
Days^2 ($\times 10^{-6}$)			-.167**	2.87***		2.07
			(.0651)	(.27)		(2.57)
Days^3 ($\times 10^{-9}$)				-.925***		-.67
				(.0798)		(.756)
Observations	8684	8684	8684	8684	8684	8684
R-square	0.369	0.369	0.369	0.379	0.577	0.577
Y-mean	84.22	84.22	84.22	84.22	84.22	84.22

Y-std.dev.	325.0	325.0	325.0	325.0	325.0	325.0
Y-mean Wave1	263.6	263.6	263.6	263.6	263.6	263.6
Y-std.dev. Wave1	600.5	600.5	600.5	600.5	600.5	600.5
Y-mean Wave2	72.14	72.14	72.14	72.14	72.14	72.14
Y-std.dev. Wave2	125.9	125.9	125.9	125.9	125.9	125.9
Y-mean Wave3	0	0	0	0	0	0
Y-std.dev. Wave3	0	0	0	0	0	0
Y-mean Wave4	1.147	1.147	1.147	1.147	1.147	1.147
Y-std.dev. Wave4	2.213	2.213	2.213	2.213	2.213	2.213
DOW FEs	Y	Y	Y	Y	Y	Y
Wave-Month FEs	Y	Y	Y	Y		
Wave-Year FEs	Y	Y	Y	Y		
Wave-Year-Month FEs					Y	Y

Notes: * significant 10% level; ** significant at 5% level; *** significant at 1% level.

Table S5: First stage Nov 14, 2016-2017

	Panel A: #All tweets (z)					
	(1)	(2)	(3)	(4)	(5)	(6)
Holiday_RU × Wave=1	-0.097	-0.103	-0.104	-0.104	-0.103	-0.103
	(0.179)	(0.179)	(0.179)	(0.179)	(0.179)	(0.179)
Holiday_RU × Wave=2	-0.110	-0.116	-0.116	-0.117	-0.112	-0.113
	(0.179)	(0.179)	(0.179)	(0.179)	(0.179)	(0.179)
Holiday_RU × Wave=3	0.006	0.000	-0.000	-0.001	0.006	0.006
	(0.179)	(0.179)	(0.179)	(0.179)	(0.179)	(0.179)
Holiday_RU × Wave=4	0.003	-0.003	-0.004	-0.004	0.003	0.002
	(0.179)	(0.179)	(0.179)	(0.179)	(0.179)	(0.179)
Temperature × Wave=1 (z)	-.0386	-.0364	-.0505	-.0505	-.0408	-.061
	(.0562)	(.0563)	(.0567)	(.0567)	(.0563)	(.0571)
Temperature × Wave=2 (z)	-.0236	-.0214	-.0355	-.0355	-.0247	-.0449
	(.0562)	(.0563)	(.0567)	(.0567)	(.0563)	(.0571)
Temperature × Wave=3 (z)	.0123	.0145	.000345	.000375	.0122	-.00801
	(.0562)	(.0563)	(.0567)	(.0567)	(.0563)	(.0571)
Temperature × Wave=4 (z)	.0118	.014	-.000154	-.000124	.0117	-.00851
	(.0562)	(.0563)	(.0567)	(.0567)	(.0563)	(.0571)
Holiday_US	-.129*	-.13*	-.129*	-.133*	-.13*	-.132*
	(.0743)	(.0744)	(.0743)	(.0744)	(.0744)	(.0744)
Days ($\times 10^{-3}$)		-.844	30.6*	466		528
		(1.41)	(16)	(456)		(458)
Days^2 ($\times 10^{-6}$)			-7.81**	-228		-252
			(3.96)	(230)		(231)
Days^3 ($\times 10^{-9}$)				36.9		39.7

				(38.6)		(38.6)
Observations	1652	1652	1652	1652	1652	1652
R-square	0.769	0.769	0.769	0.769	0.769	0.770
Y-mean	310.3	310.3	310.3	310.3	310.3	310.3
Y-std.dev.	667.3	667.3	667.3	667.3	667.3	667.3
Y-mean Wave1	1001.1	1001.1	1001.1	1001.1	1001.1	1001.1
Y-std.dev. Wave1	1040.8	1040.8	1040.8	1040.8	1040.8	1040.8
Y-mean Wave2	234.7	234.7	234.7	234.7	234.7	234.7
Y-std.dev. Wave2	164.4	164.4	164.4	164.4	164.4	164.4
Y-mean Wave3	0	0	0	0	0	0
Y-std.dev. Wave3	0	0	0	0	0	0
Y-mean Wave4	5.310	5.310	5.310	5.310	5.310	5.310
Y-std.dev. Wave4	2.679	2.679	2.679	2.679	2.679	2.679
	Panel B: #Original tweets (z)					
Holiday_RU × Wave=1	-0.275	-0.272	-0.273	-0.273	-0.275	-0.266
	(0.173)	(0.174)	(0.173)	(0.173)	(0.173)	(0.173)
Holiday_RU × Wave=2	-0.056	-0.054	-0.055	-0.055	-0.059	-0.051
	(0.173)	(0.174)	(0.173)	(0.173)	(0.173)	(0.173)
Holiday_RU × Wave=3	0.026	0.028	0.028	0.027	0.026	0.035
	(0.173)	(0.174)	(0.173)	(0.173)	(0.173)	(0.173)
Holiday_RU × Wave=4	0.019	0.021	0.021	0.020	0.019	0.028
	(0.173)	(0.174)	(0.173)	(0.173)	(0.173)	(0.173)
Temperature × Wave=1 (z)	.197***	.196***	.182***	.182***	.197***	.168***
	(.0544)	(.0546)	(.055)	(.0549)	(.0546)	(.0553)
Temperature × Wave=2 (z)	.00728	.00657	-.00791	-.00787	.00607	-.0226
	(.0544)	(.0546)	(.055)	(.0549)	(.0546)	(.0553)
Temperature × Wave=3 (z)	.0161	.0154	.000896	.00094	.0161	-.0125
	(.0544)	(.0546)	(.055)	(.0549)	(.0546)	(.0553)
Temperature × Wave=4 (z)	.015	.0143	-.000202	-.000158	.015	-.0137
	(.0544)	(.0546)	(.055)	(.0549)	(.0546)	(.0553)
Holiday_US	-.082	-.0819	-.0813	-.0865	-.082	-.0858
	(.0721)	(.0721)	(.072)	(.0721)	(.0722)	(.0721)
Days ($\times 10^{-3}$)		.27	32.4**	687		780*
		(1.37)	(15.5)	(442)		(444)
Days^2 ($\times 10^{-6}$)			-7.99**	-338		-374*
			(3.84)	(223)		(224)
Days^3 ($\times 10^{-9}$)				55.4		59.7
				(37.4)		(37.4)
Observations	1652	1652	1652	1652	1652	1652
R-square	0.782	0.782	0.783	0.783	0.782	0.784
Y-mean	106.7	106.7	106.7	106.7	106.7	106.7
Y-std.dev.	225.2	225.2	225.2	225.2	225.2	225.2
Y-mean Wave1	377.6	377.6	377.6	377.6	377.6	377.6
Y-std.dev. Wave1	319.2	319.2	319.2	319.2	319.2	319.2

Y-mean Wave2	44.75	44.75	44.75	44.75	44.75	44.75
Y-std.dev. Wave2	44.60	44.60	44.60	44.60	44.60	44.60
Y-mean Wave3	0	0	0	0	0	0
Y-std.dev. Wave3	0	0	0	0	0	0
Y-mean Wave4	4.513	4.513	4.513	4.513	4.513	4.513
Y-std.dev. Wave4	2.415	2.415	2.415	2.415	2.415	2.415
	Panel C: #Retweeted tweets (z)					
Holiday_RU × Wave=1	-0.006	-0.016	-0.016	-0.017	-0.014	-0.019
	(0.198)	(0.199)	(0.199)	(0.199)	(0.199)	(0.199)
Holiday_RU × Wave=2	-0.127	-0.136	-0.137	-0.137	-0.129	-0.134
	(0.198)	(0.199)	(0.199)	(0.199)	(0.199)	(0.199)
Holiday_RU × Wave=3	-0.003	-0.013	-0.014	-0.014	-0.004	-0.009
	(0.198)	(0.199)	(0.199)	(0.199)	(0.199)	(0.199)
Holiday_RU × Wave=4	-0.005	-0.015	-0.015	-0.015	-0.005	-0.010
	(0.198)	(0.199)	(0.199)	(0.199)	(0.199)	(0.199)
Temperature × Wave=1 (z)	-.147**	-.143**	-.156**	-.156**	-.15**	-.164***
	(.0624)	(.0625)	(.063)	(.063)	(.0625)	(.0635)
Temperature × Wave=2 (z)	-.0364	-.033	-.0459	-.0459	-.0373	-.052
	(.0624)	(.0625)	(.063)	(.063)	(.0625)	(.0635)
Temperature × Wave=3 (z)	.00961	.013	.00006	.0000804	.00944	-.00529
	(.0624)	(.0625)	(.063)	(.063)	(.0625)	(.0635)
Temperature × Wave=4 (z)	.00943	.0128	-.00012	-.0000994	.00928	-.00545
	(.0624)	(.0625)	(.063)	(.063)	(.0625)	(.0635)
Holiday_US	-.142*	-.143*	-.142*	-.145*	-.142*	-.144*
	(.0826)	(.0826)	(.0826)	(.0827)	(.0827)	(.0827)
Days ($\times 10^{-3}$)		-1.31	27.4	328		370
		(1.57)	(17.8)	(507)		(510)
Days^2 ($\times 10^{-6}$)			-7.15	-159		-175
			(4.4)	(256)		(257)
Days^3 ($\times 10^{-9}$)				25.4		27.4
				(42.9)		(43)
Observations	1652	1652	1652	1652	1652	1652
R-square	0.714	0.714	0.715	0.715	0.715	0.715
Y-mean	203.6	203.6	203.6	203.6	203.6	203.6
Y-std.dev.	477.6	477.6	477.6	477.6	477.6	477.6
Y-mean Wave1	623.5	623.5	623.5	623.5	623.5	623.5
Y-std.dev. Wave1	797.3	797.3	797.3	797.3	797.3	797.3
Y-mean Wave2	189.9	189.9	189.9	189.9	189.9	189.9
Y-std.dev. Wave2	137.3	137.3	137.3	137.3	137.3	137.3
Y-mean Wave3	0	0	0	0	0	0
Y-std.dev. Wave3	0	0	0	0	0	0
Y-mean Wave4	0.7966	0.7966	0.7966	0.7966	0.7966	0.7966
Y-std.dev. Wave4	1.214	1.214	1.214	1.214	1.214	1.214
DOW FEs	Y	Y	Y	Y	Y	Y

Wave-Month FEs	Y	Y	Y	Y		
Wave-Year FEs	Y	Y	Y	Y		
Wave-Year-Month FEs					Y	Y

Notes: * significant 10% level; ** significant at 5% level; *** significant at 1% level.

S1.5 Adding other weather variables

Table S6: First stage: Russian holiday and temperature on blocked tweets on the day shift

	Panel A: #All tweets (z)					
	(1)	(2)	(3)	(4)	(5)	(6)
Holiday_RU × Wave=1	-0.362***	-0.361***	-0.362***	-0.363***	-0.371***	-0.375***
	(0.116)	(0.116)	(0.116)	(0.116)	(0.105)	(0.105)
Holiday_RU × Wave=2	-0.047	-0.046	-0.047	-0.048	-0.046	-0.050
	(0.116)	(0.116)	(0.116)	(0.116)	(0.105)	(0.105)
Holiday_RU × Wave=3	-0.007	-0.007	-0.008	-0.009	-0.007	-0.010
	(0.116)	(0.116)	(0.116)	(0.116)	(0.105)	(0.105)
Holiday_RU × Wave=4	-0.010	-0.009	-0.010	-0.011	-0.010	-0.013
	(0.116)	(0.116)	(0.116)	(0.116)	(0.105)	(0.105)
Temperature × Wave=1 (z)	.0556	.0556	.0579	.0593	-.00239	-.00218
	(.0372)	(.0372)	(.0372)	(.0372)	(.0389)	(.0389)
Temperature × Wave=2 (z)	-.00882	-.00884	-.00654	-.00512	.00761	.00782
	(.0372)	(.0372)	(.0372)	(.0372)	(.0389)	(.0389)
Temperature × Wave=3 (z)	.0073	.00728	.00959	.011	.00424	.00446
	(.0372)	(.0372)	(.0372)	(.0372)	(.0389)	(.0389)
Temperature × Wave=4 (z)	.00458	.00456	.00686	.00828	.00237	.00258
	(.0372)	(.0372)	(.0372)	(.0372)	(.0389)	(.0389)
Snow or ice (dummy)	-.00662	-.00665	-.00107	.0064	-.0304	-.0303
	(.0262)	(.0262)	(.0262)	(.0264)	(.0247)	(.0248)
Cloud coverage	-.0985***	-.0984***	-.0982***	-.103***	-.0698**	-.0701**
	(.0345)	(.0345)	(.0345)	(.0346)	(.0324)	(.0324)
Wind speed (knot)	.00401	.00401	.00374	.00402	.00146	.00146
	(.00324)	(.00324)	(.00324)	(.00324)	(.00302)	(.00303)
Precipitation (inch)	.0601	.06	.0556	.0541	.0511	.0516
	(.0425)	(.0425)	(.0425)	(.0425)	(.0394)	(.0394)
Holiday_US	.0155	.0156	.0141	.0149	.00975	.00942
	(.0532)	(.0532)	(.0532)	(.0532)	(.0481)	(.0481)
Days ($\times 10^{-3}$)		.12	.56	1.03		-.68
		(.96)	(.969)	(.987)		(2.87)
Days^2 ($\times 10^{-6}$)			-.156***	-.612***		.0604
			(.047)	(.192)		(2.11)

Days^3 ($\times 10^{-9}$)				.103**		-.00273
				(.0419)		(.453)
Observations	10076	10076	10076	10076	10076	10076
R-square	0.372	0.372	0.373	0.373	0.506	0.506
Y-mean	185.2	185.2	185.2	185.2	185.2	185.2
Y-std.dev.	722.9	722.9	722.9	722.9	722.9	722.9
Y-mean Wave1	628.7	628.7	628.7	628.7	628.7	628.7
Y-std.dev. Wave1	1339.1	1339.1	1339.1	1339.1	1339.1	1339.1
Y-mean Wave2	106.4	106.4	106.4	106.4	106.4	106.4
Y-std.dev. Wave2	166.3	166.3	166.3	166.3	166.3	166.3
Y-mean Wave3	0.0012	0.0012	0.0012	0.0012	0.0012	0.0012
Y-std.dev. Wave3	0.0445	0.0445	0.0445	0.0445	0.0445	0.0445
Y-mean Wave4	5.644	5.644	5.644	5.644	5.644	5.644
Y-std.dev. Wave4	20.31	20.31	20.31	20.31	20.31	20.31
	Panel B: #Original tweets (z)					
Holiday_RU × Wave=1	-0.324***	-0.324***	-0.324***	-0.327***	-0.332***	-0.336***
	(0.123)	(0.123)	(0.123)	(0.123)	(0.113)	(0.114)
Holiday_RU × Wave=2	-0.024	-0.024	-0.024	-0.027	-0.024	-0.028
	(0.123)	(0.123)	(0.123)	(0.123)	(0.113)	(0.114)
Holiday_RU × Wave=3	-0.005	-0.004	-0.005	-0.007	-0.004	-0.008
	(0.123)	(0.123)	(0.123)	(0.123)	(0.113)	(0.114)
Holiday_RU × Wave=4	-0.007	-0.007	-0.007	-0.009	-0.007	-0.011
	(0.123)	(0.123)	(0.123)	(0.123)	(0.113)	(0.114)
Temperature × Wave=1 (z)	.0984**	.0984**	.0995**	.103***	.028	.0283
	(.0394)	(.0394)	(.0394)	(.0394)	(.0421)	(.0421)
Temperature × Wave=2 (z)	.00992	.00991	.011	.0141	.0143	.0147
	(.0394)	(.0394)	(.0394)	(.0394)	(.0421)	(.0421)
Temperature × Wave=3 (z)	.00306	.00305	.00415	.00725	.00378	.0041
	(.0394)	(.0394)	(.0394)	(.0394)	(.0421)	(.0421)
Temperature × Wave=4 (z)	-.0000314	-.0000436	.00106	.00416	.00143	.00175
	(.0394)	(.0394)	(.0394)	(.0394)	(.0421)	(.0421)
Snow or ice (dummy)	-.036	-.0361	-.0334	-.0171	-.0373	-.0372
	(.0277)	(.0277)	(.0278)	(.0279)	(.0268)	(.0268)
Cloud coverage	-.0902**	-.0901**	-.09**	-.101***	-.0713**	-.0715**
	(.0366)	(.0366)	(.0366)	(.0366)	(.0351)	(.0351)
Wind speed (knot)	-.00304	-.00303	-.00316	-.00255	-.00373	-.00375
	(.00343)	(.00343)	(.00343)	(.00343)	(.00327)	(.00328)
Precipitation (inch)	.0899**	.0898**	.0877*	.0845*	.071*	.0719*
	(.045)	(.045)	(.045)	(.045)	(.0426)	(.0427)
Holiday_US	.0328	.0329	.0322	.0338	.0281	.0277
	(.0564)	(.0564)	(.0564)	(.0563)	(.052)	(.052)
Days ($\times 10^{-3}$)		.0728	.284	1.3		-.335
		(1.02)	(1.03)	(1.04)		(3.11)
Days^2 ($\times 10^{-6}$)			-.0748	-1.07***		-.364

			(.0497)	(.203)		(2.28)
Days^3 ($\times 10^{-9}$)				.224***		.103
				(.0443)		(.49)
Observations	10076	10076	10076	10076	10076	10076
R-square	0.294	0.294	0.295	0.296	0.420	0.420
Y-mean	108.4	108.4	108.4	108.4	108.4	108.4
Y-std.dev.	592.8	592.8	592.8	592.8	592.8	592.8
Y-mean Wave1	405.4	405.4	405.4	405.4	405.4	405.4
Y-std.dev. Wave1	1133.2	1133.2	1133.2	1133.2	1133.2	1133.2
Y-mean Wave2	23.78	23.78	23.78	23.78	23.78	23.78
Y-std.dev. Wave2	58.40	58.40	58.40	58.40	58.40	58.40
Y-mean Wave3	0.0012	0.0012	0.0012	0.0012	0.0012	0.0012
Y-std.dev. Wave3	0.0445	0.0445	0.0445	0.0445	0.0445	0.0445
Y-mean Wave4	4.297	4.297	4.297	4.297	4.297	4.297
Y-std.dev. Wave4	19.93	19.93	19.93	19.93	19.93	19.93
	Panel C: #Retweeted tweets (z)					
Holiday_RU × Wave=1	-0.231**	-0.230*	-0.232**	-0.230*	-0.237**	-0.239**
	(0.117)	(0.118)	(0.118)	(0.117)	(0.097)	(0.098)
Holiday_RU × Wave=2	-0.066	-0.065	-0.066	-0.064	-0.064	-0.066
	(0.117)	(0.118)	(0.118)	(0.117)	(0.097)	(0.098)
Holiday_RU × Wave=3	-0.008	-0.007	-0.009	-0.007	-0.008	-0.010
	(0.117)	(0.118)	(0.118)	(0.117)	(0.097)	(0.098)
Holiday_RU × Wave=4	-0.010	-0.009	-0.010	-0.008	-0.010	-0.012
	(0.117)	(0.118)	(0.118)	(0.117)	(0.097)	(0.098)
Temperature × Wave=1 (z)	-.0608	-.0609	-.0575	-.0602	-.0613*	-.0615*
	(.0377)	(.0377)	(.0377)	(.0376)	(.0362)	(.0362)
Temperature × Wave=2 (z)	-.041	-.041	-.0377	-.0404	-.0101	-.0102
	(.0377)	(.0377)	(.0377)	(.0376)	(.0362)	(.0362)
Temperature × Wave=3 (z)	.0116	.0116	.0149	.0122	.00276	.00264
	(.0377)	(.0377)	(.0377)	(.0376)	(.0362)	(.0362)
Temperature × Wave=4 (z)	.0111	.0111	.0145	.0118	.00287	.00275
	(.0377)	(.0377)	(.0377)	(.0376)	(.0362)	(.0362)
Snow or ice (dummy)	.0555**	.0555**	.0637**	.0494*	.000484	.000507
	(.0265)	(.0265)	(.0266)	(.0267)	(.023)	(.023)
Cloud coverage	-.0592*	-.0591*	-.0588*	-.0493	-.0272	-.0274
	(.035)	(.035)	(.035)	(.035)	(.0302)	(.0302)
Wind speed (knot)	.0157***	.0157***	.0153***	.0148***	.0109***	.011***
	(.00329)	(.00329)	(.00328)	(.00328)	(.00281)	(.00281)
Precipitation (inch)	-.0331	-.0333	-.0398	-.0369	-.0174	-.0178
	(.043)	(.0431)	(.043)	(.043)	(.0366)	(.0367)
Holiday_US	-.0276	-.0275	-.0296	-.0311	-.0321	-.0322
	(.0539)	(.0539)	(.0539)	(.0538)	(.0447)	(.0447)
Days ($\times 10^{-3}$)		.146	.791	-.104		-.979
		(.973)	(.981)	(.999)		(2.67)

Days^2 ($\times 10^{-6}$)			-.229***	.647***		.868
			(.0476)	(.194)		(1.96)
Days^3 ($\times 10^{-9}$)				-.197***		-.211
				(.0424)		(.421)
Observations	10076	10076	10076	10076	10076	10076
R-square	0.374	0.374	0.376	0.377	0.585	0.585
Y-mean	76.81	76.81	76.81	76.81	76.81	76.81
Y-std.dev.	303.5	303.5	303.5	303.5	303.5	303.5
Y-mean Wave1	223.3	223.3	223.3	223.3	223.3	223.3
Y-std.dev. Wave1	563.4	563.4	563.4	563.4	563.4	563.4
Y-mean Wave2	82.64	82.64	82.64	82.64	82.64	82.64
Y-std.dev. Wave2	134.2	134.2	134.2	134.2	134.2	134.2
Y-mean Wave3	0	0	0	0	0	0
Y-std.dev. Wave3	0	0	0	0	0	0
Y-mean Wave4	1.347	1.347	1.347	1.347	1.347	1.347
Y-std.dev. Wave4	2.689	2.689	2.689	2.689	2.689	2.689
DOW FEs	Y	Y	Y	Y	Y	Y
Wave-Month FEs	Y	Y	Y	Y		
Wave-Year FEs	Y	Y	Y	Y		
Wave-Year-Month FEs					Y	Y

Notes: The sample size is smaller than that in Table 1 is due to missing weather data. * significant 10% level; ** significant at 5% level; *** significant at 1% level.

S1.6 Holiday week January 1-7

Table S7: First stage: Russian holiday and temperature on blocked tweets on the day shift

	Panel A: #All tweets (z)					
	(1)	(2)	(3)	(4)	(5)	(6)
Y-mean	166.8	166.8	166.8	166.8	166.8	166.8
Y-std.dev.	687.8	687.8	687.8	687.8	687.8	687.8
Y-mean Wave1	568.4	568.4	568.4	568.4	568.4	568.4
Y-std.dev. Wave1	1283.1	1283.1	1283.1	1283.1	1283.1	1283.1
Y-mean Wave2	93.16	93.16	93.16	93.16	93.16	93.16
Y-std.dev. Wave2	159.1	159.1	159.1	159.1	159.1	159.1
Y-mean Wave3	0.0010	0.0010	0.0010	0.0010	0.0010	0.0010
Y-std.dev. Wave3	0.0416	0.0416	0.0416	0.0416	0.0416	0.0416
Y-mean Wave4	5.801	5.801	5.801	5.801	5.801	5.801
Y-std.dev. Wave4	19.38	19.38	19.38	19.38	19.38	19.38
	Panel B: #Original tweets (z)					
Y-mean	98.98	98.98	98.98	98.98	98.98	98.98

Y-std.dev.	563.6	563.6	563.6	563.6	563.6	563.6
Y-mean Wave1	370.4	370.4	370.4	370.4	370.4	370.4
Y-std.dev. Wave1	1081.3	1081.3	1081.3	1081.3	1081.3	1081.3
Y-mean Wave2	21.04	21.04	21.04	21.04	21.04	21.04
Y-std.dev. Wave2	55.05	55.05	55.05	55.05	55.05	55.05
Y-mean Wave3	0.0010	0.0010	0.0010	0.0010	0.0010	0.0010
Y-std.dev. Wave3	0.0416	0.0416	0.0416	0.0416	0.0416	0.0416
Y-mean Wave4	4.449	4.449	4.449	4.449	4.449	4.449
Y-std.dev. Wave4	18.92	18.92	18.92	18.92	18.92	18.92
	Panel C: #Retweeted tweets (z)					
Y-mean	67.87	67.87	67.87	67.87	67.87	67.87
Y-std.dev.	285.6	285.6	285.6	285.6	285.6	285.6
Y-mean Wave1	198.0	198.0	198.0	198.0	198.0	198.0
Y-std.dev. Wave1	532.7	532.7	532.7	532.7	532.7	532.7
Y-mean Wave2	72.11	72.11	72.11	72.11	72.11	72.11
Y-std.dev. Wave2	128.2	128.2	128.2	128.2	128.2	128.2
Y-mean Wave3	0	0	0	0	0	0
Y-std.dev. Wave3	0	0	0	0	0	0
Y-mean Wave4	1.351	1.351	1.351	1.351	1.351	1.351
Y-std.dev. Wave4	2.663	2.663	2.663	2.663	2.663	2.663
DOW FEs	Y	Y	Y	Y	Y	Y
Wave-Month FEs	Y	Y	Y	Y		
Wave-Year FEs	Y	Y	Y	Y		
Wave-Year-Month FEs					Y	Y

Notes: We code the whole holiday week January 1-7 as `Holiday_RU` and replicate Table 1. Holiday effects are still negative and significant, but magnitudes decrease by half. The sample size is smaller than that in Table 1 is due to missing weather data. * significant 10% level; ** significant at 5% level; *** significant at 1% level.

S2 Holiday Event Study (Regression Tables)

Here we focus on the 21 days around the Russian holiday. That is, we consider the subsample of 10 days prior to the holiday, the holiday itself, and 10 days after the holiday. We use 5 days prior and after the holiday as robustness check to prevent overlap in any two holiday events. We also drop January 1 and January 7 given the holiday week concern.

S2.1 First stage

Table S8: First stage, drop 10 busiest days, 42, 42, 44 events 2012-2017

	Panel A: #All tweets (z)					
	(1)	(2)	(3)	(4)	(5)	(6)
Holiday_RU	-0.203***	-0.214***	-0.214***	-0.212***	-0.200***	-0.214***
	(0.073)	(0.075)	(0.075)	(0.074)	(0.071)	(0.075)
Holiday_US	-0.097	-0.106	-0.108	-0.117	-0.131	-0.141
	(0.102)	(0.105)	(0.100)	(0.098)	(0.109)	(0.110)
Days ($\times 10^{-3}$)		-4.422	-2.708	-7.519		-4.814
		(7.032)	(7.086)	(6.802)		(10.186)
Days^2 ($\times 10^{-6}$)			-0.774	5.902***		-0.168
			(0.544)	(2.027)		(19.800)
Days^3 ($\times 10^{-9}$)				-2.04***		.00514
				(.613)		(6.09)
Observations	882	882	882	882	882	882
R-square	0.525	0.526	0.535	0.567	0.704	0.704
Y-mean	605.7	605.7	605.7	605.7	605.7	605.7
Y-std.dev.	839.6	839.6	839.6	839.6	839.6	839.6
	Panel B: #Original tweets (z)					
Holiday_RU	-0.184**	-0.187**	-0.187**	-0.185**	-0.183**	-0.185**
	(0.074)	(0.079)	(0.079)	(0.078)	(0.074)	(0.080)
Holiday_US	-0.038	-0.041	-0.045	-0.051	-0.047	-0.051
	(0.115)	(0.114)	(0.111)	(0.108)	(0.113)	(0.111)
Days ($\times 10^{-3}$)		-1.254	1.501	-2.020		-3.342
		(4.896)	(4.862)	(5.359)		(7.905)
Days^2 ($\times 10^{-6}$)			-1.244***	3.642		-0.455
			(0.385)	(3.262)		(15.906)
Days^3 ($\times 10^{-9}$)				-1.49		.725
				(1.03)		(4.89)
Observations	882	882	882	882	882	882
R-square	0.495	0.495	0.520	0.537	0.655	0.656
Y-mean	323.2	323.2	323.2	323.2	323.2	323.2
Y-std.dev.	477.4	477.4	477.4	477.4	477.4	477.4
	Panel C: #Retweeted tweets (z)					

Holiday_RU	-0.164*	-0.180*	-0.181*	-0.178*	-0.165*	-0.183*
	(0.091)	(0.100)	(0.100)	(0.100)	(0.091)	(0.100)
Holiday_US	-0.058	-0.077	-0.076	-0.071	-0.063	-0.076
	(0.085)	(0.090)	(0.090)	(0.090)	(0.090)	(0.097)
Days ($\times 10^{-3}$)		-6.777	-6.030	-9.755		-5.211
		(13.293)	(13.191)	(13.213)		(18.976)
Days^2 ($\times 10^{-6}$)			-0.348	5.382***		2.225
			(0.814)	(1.729)		(37.214)
Days^3 ($\times 10^{-9}$)				-1.84***		-1.58
				(.594)		(11.3)
Observations	924	924	924	924	924	924
R-square	0.364	0.365	0.367	0.393	0.613	0.615
Y-mean	208.0	208.0	208.0	208.0	208.0	208.0
Y-std.dev.	434.8	434.8	434.8	434.8	434.8	434.8
DOW FEs	Y	Y	Y	Y	Y	Y
Month FEs	Y	Y	Y	Y		
Year FEs	Y	Y	Y	Y		
Year-Month FEs					Y	Y

Notes: We drop top 10 busiest days for each category (all, original, retweeted tweets) 2012-2017, and only keep holiday events with complete data over the 21-day window. This results in a smaller number of events than 48 (8 events per year over 6 years) and different number of events for each category, equal to #observations over 21. Standard errors are clustered at the event level. * significant 10% level; ** significant at 5% level; *** significant at 1% level.

Table S9: First stage, 46 events 2012-2017

	Panel A: #All tweets (z)					
	(1)	(2)	(3)	(4)	(5)	(6)
Holiday_RU	-0.191***	-0.166***	-0.166***	-0.166***	-0.192***	-0.166***
	(0.061)	(0.053)	(0.053)	(0.053)	(0.062)	(0.054)
Holiday_US	-0.215	-0.191	-0.193	-0.194	-0.218	-0.174
	(0.162)	(0.166)	(0.166)	(0.166)	(0.166)	(0.171)
Days ($\times 10^{-3}$)		10.197	11.338	10.379		-24.951
		(12.688)	(12.749)	(13.109)		(21.420)
Days^2 ($\times 10^{-6}$)			-0.522**	0.813		46.236
			(0.254)	(1.568)		(43.414)
Days^3 ($\times 10^{-9}$)				-.411		-14.1
				(.473)		(13.2)
Observations	966	966	966	966	966	966
R-square	0.250	0.252	0.256	0.257	0.391	0.397
Y-mean	832.7	832.7	832.7	832.7	832.7	832.7
Y-std.dev.	1672.5	1672.5	1672.5	1672.5	1672.5	1672.5

	Panel B: #Original tweets (z)					
Holiday_RU	-0.150**	-0.118**	-0.118**	-0.119**	-0.152**	-0.118**
	(0.058)	(0.048)	(0.048)	(0.048)	(0.060)	(0.048)
Holiday_US	-0.205	-0.175	-0.177	-0.176	-0.194	-0.143
	(0.175)	(0.178)	(0.178)	(0.178)	(0.178)	(0.182)
Days ($\times 10^{-3}$)		12.718	13.915	14.258		-24.887
		(13.216)	(13.292)	(13.636)		(21.947)
Days^2 ($\times 10^{-6}$)			-0.547***	-1.025		48.717
			(0.167)	(1.575)		(44.620)
Days^3 ($\times 10^{-9}$)				.147		-14.6
				(.478)		(13.5)
Observations	966	966	966	966	966	966
R-square	0.205	0.208	0.212	0.213	0.359	0.367
Y-mean	572.9	572.9	572.9	572.9	572.9	572.9
Y-std.dev.	1571.5	1571.5	1571.5	1571.5	1571.5	1571.5
	Panel C: #Retweeted tweets (z)					
Holiday_RU	-0.163**	-0.177**	-0.177**	-0.174**	-0.158**	-0.175**
	(0.075)	(0.082)	(0.082)	(0.081)	(0.073)	(0.081)
Holiday_US	-0.072	-0.086	-0.086	-0.091	-0.115	-0.128
	(0.089)	(0.094)	(0.093)	(0.089)	(0.103)	(0.107)
Days ($\times 10^{-3}$)		-5.611	-5.559	-9.666		-5.022
		(10.823)	(10.877)	(10.949)		(15.846)
Days^2 ($\times 10^{-6}$)			-0.024	5.689***		1.485
			(0.655)	(1.721)		(30.991)
Days^3 ($\times 10^{-9}$)				-1.76***		-1.09
				(.538)		(9.36)
Observations	966	966	966	966	966	966
R-square	0.389	0.390	0.390	0.417	0.643	0.644
Y-mean	259.7	259.7	259.7	259.7	259.7	259.7
Y-std.dev.	522.2	522.2	522.2	522.2	522.2	522.2
DOW FEs	Y	Y	Y	Y	Y	Y
Month FEs	Y	Y	Y	Y		
Year FEs	Y	Y	Y	Y		
Year-Month FEs					Y	Y

Notes: Standard errors are clustered at the event level. * significant 10% level; ** significant at 5% level; *** significant at 1% level.

Table S10: First stage, dynamic event time

	Panel A: #All tweets (z)					
	(1)	(2)	(3)	(4)	(5)	(6)
Holiday_RU Day -8,-7	-0.023	-0.021	-0.021	-0.021	-0.019	-0.016
	(0.064)	(0.066)	(0.070)	(0.069)	(0.059)	(0.058)
Holiday_RU -6,-5	0.044	0.056	0.055	0.054	0.046	0.061
	(0.092)	(0.099)	(0.104)	(0.106)	(0.084)	(0.080)
Holiday_RU -4,-3	-0.069	-0.049	-0.049	-0.050	-0.067	-0.042
	(0.073)	(0.093)	(0.099)	(0.096)	(0.061)	(0.053)
Holiday_RU -2,-1	-0.072	-0.042	-0.043	-0.045	-0.072	-0.035
	(0.086)	(0.114)	(0.119)	(0.113)	(0.076)	(0.068)
Holiday_RU	-.196*	-.201*	-.201**	-.198*	-.192*	-.198*
	(.101)	(.1)	(.0992)	(.0983)	(.0992)	(.101)
Holiday_RU 1,2	-.0603	-.026	-.0278	-.0294	-.0592	-.0157
	(.118)	(.15)	(.153)	(.141)	(.0982)	(.0806)
Holiday_RU 3,4	.0427	.0879	.087	.085	.044	.101
	(.0909)	(.137)	(.14)	(.122)	(.0836)	(.0658)
Holiday_RU 5,6	-.0374	.0168	.0156	.014	-.0389	.03
	(.11)	(.171)	(.175)	(.155)	(.0952)	(.0689)
Holiday_RU 7,8	-.0696	-.00877	-.0106	-.0129	-.0662	.0113
	(.134)	(.192)	(.198)	(.176)	(.122)	(.0829)
Holiday_RU 9,10	-.0605	.0107	.00922	.00551	-.0578	.033
	(.148)	(.228)	(.234)	(.205)	(.13)	(.0898)
Holiday_US	-.0835	-.0887	-.0909	-.0992	-.119	-.126
	(.108)	(.111)	(.106)	(.103)	(.117)	(.116)
Days ($\times 10^{-3}$)		-5.45	-3.67	-8.3		-5.82
		(10.9)	(11.2)	(9.55)		(11.7)
Days^2 ($\times 10^{-6}$)			-.783	5.84***		-.995
			(.543)	(2.03)		(18.8)
Days^3 ($\times 10^{-9}$)				-2.03***		.216
				(.613)		(5.82)
Observations	882	882	882	882	882	882
R-square	0.529	0.529	0.539	0.570	0.706	0.707
Y-mean	605.7	605.7	605.7	605.7	605.7	605.7
Y-std.dev.	839.6	839.6	839.6	839.6	839.6	839.6
	Panel B: #Original tweets (z)					
Holiday_RU Day -8,-7	-0.019	-0.016	-0.017	-0.017	-0.019	-0.018
	(0.045)	(0.044)	(0.043)	(0.042)	(0.055)	(0.055)
Holiday_RU -6,-5	0.017	0.027	0.027	0.025	0.016	0.024
	(0.093)	(0.090)	(0.088)	(0.090)	(0.092)	(0.086)
Holiday_RU -4,-3	0.028	0.045	0.045	0.044	0.027	0.043
	(0.070)	(0.071)	(0.070)	(0.069)	(0.077)	(0.072)
Holiday_RU -2,-1	-0.021	0.004	0.002	0.001	-0.023	-0.001

	(0.075)	(0.077)	(0.079)	(0.079)	(0.078)	(0.077)
Holiday_RU	-.168**	-.172**	-.172**	-.17**	-.167**	-.17**
	(.0759)	(.0775)	(.0767)	(.0761)	(.075)	(.0779)
Holiday_RU 1,2	.0575	.0874	.0846	.0834	.056	.0816
	(.086)	(.0939)	(.0951)	(.0953)	(.0783)	(.0668)
Holiday_RU 3,4	.113	.153	.151	.15	.112	.146
	(.132)	(.129)	(.131)	(.131)	(.135)	(.118)
Holiday_RU 5,6	.137	.184	.182	.181	.133	.173
	(.115)	(.13)	(.132)	(.13)	(.112)	(.103)
Holiday_RU 7,8	.0225	.0756	.0727	.071	.0208	.0653
	(.0849)	(.105)	(.113)	(.107)	(.0811)	(.0653)
Holiday_RU 9,10	-.0534	.0087	.00632	.00361	-.0549	-.00291
	(.105)	(.122)	(.131)	(.121)	(.108)	(.0939)
Holiday_US	-.000829	-.00535	-.00897	-.015	-.00981	-.0155
	(.119)	(.119)	(.116)	(.113)	(.117)	(.117)
Days ($\times 10^{-3}$)		-4.76	-1.91	-5.31		-6.74
		(6.5)	(6.72)	(6.06)		(8.43)
Days^2 ($\times 10^{-6}$)			-1.25***	3.6		.168
			(.384)	(3.27)		(15.2)
Days^3 ($\times 10^{-9}$)				-1.48		.523
				(1.04)		(4.67)
Observations	882	882	882	882	882	882
R-square	0.499	0.499	0.524	0.541	0.659	0.659
Y-mean	323.2	323.2	323.2	323.2	323.2	323.2
Y-std.dev.	477.4	477.4	477.4	477.4	477.4	477.4

	Panel C: #Retweeted tweets (z)					
Holiday_RU Day -8,-7	-0.039	-0.037	-0.037	-0.038	-0.039	-0.035
	(0.054)	(0.054)	(0.055)	(0.053)	(0.058)	(0.055)
Holiday_RU -6,-5	-0.025	-0.014	-0.013	-0.015	-0.023	-0.008
	(0.087)	(0.088)	(0.088)	(0.086)	(0.088)	(0.079)
Holiday_RU -4,-3	-0.121	-0.101	-0.101	-0.104	-0.119	-0.094
	(0.099)	(0.110)	(0.109)	(0.101)	(0.088)	(0.066)
Holiday_RU -2,-1	-0.071	-0.041	-0.041	-0.044	-0.070	-0.032
	(0.128)	(0.149)	(0.146)	(0.134)	(0.116)	(0.092)
Holiday_RU	-.205	-.21	-.21	-.208	-.206	-.211
	(.148)	(.151)	(.151)	(.151)	(.15)	(.155)
Holiday_RU 1,2	-.179	-.143	-.144	-.147	-.178	-.133
	(.18)	(.185)	(.184)	(.172)	(.177)	(.139)
Holiday_RU 3,4	-.0668	-.0202	-.0201	-.0252	-.0643	-.00527
	(.187)	(.203)	(.201)	(.184)	(.184)	(.133)
Holiday_RU 5,6	-.185	-.129	-.13	-.135	-.183	-.113
	(.187)	(.216)	(.214)	(.191)	(.182)	(.111)
Holiday_RU 7,8	-.149	-.0866	-.0872	-.0935	-.147	-.0681
	(.222)	(.256)	(.254)	(.228)	(.216)	(.136)

Holiday_RU 9,10	-.0837	-.011	-.0112	-.0197	-.0803	.012
	(.219)	(.277)	(.274)	(.246)	(.21)	(.12)
Holiday_US	-.064	-.0708	-.0706	-.0645	-.068	-.0716
	(.0827)	(.0859)	(.0866)	(.0857)	(.0865)	(.0915)
Days ($\times 10^{-3}$)		-5.7	-4.9	-8.32		-3.72
		(15.9)	(15.7)	(14.2)		(22.2)
Days^2 ($\times 10^{-6}$)			-.366	5.36***		.583
			(.814)	(1.73)		(35.6)
Days^3 ($\times 10^{-9}$)				-1.83***		-1.08
				(.595)		(10.8)
Observations	924	924	924	924	924	924
R-square	0.370	0.370	0.371	0.398	0.618	0.619
Y-mean	208.0	208.0	208.0	208.0	208.0	208.0
Y-std.dev.	434.8	434.8	434.8	434.8	434.8	434.8
DOW FEs	Y	Y	Y	Y	Y	Y
Month FEs	Y	Y	Y	Y		
Year FEs	Y	Y	Y	Y		
Year-Month FEs					Y	Y

Notes: We conduct a dynamic event study to examine whether there are significant effects on the days before and after the holidays. We put 2 days into one bin, and the dummy for day = -10,-9 is omitted. Small and imprecise estimates on day -8 to -1 indicate no pre-treatment trends; estimates on day 1 to 10 suggest the holiday effect is short-lasting. We conclude that holiday itself rather than neighbor days decreases #tweets and our main specification is reasonable to test the holiday effect.

S2.2 Reduced form for Hedonometer and 2020 election odds

Table S11: Reduced form, 46 events 2012-2017 on hedonometer

	Hedonometer					
	(1)	(2)	(3)	(4)	(5)	(6)
Holiday_RU	0.000	-0.001	-0.001	-0.001	0.000	-0.001
	(0.004)	(0.004)	(0.004)	(0.004)	(0.005)	(0.005)
Holiday_US	0.080***	0.079***	0.078***	0.078***	0.079***	0.078***
	(0.017)	(0.016)	(0.016)	(0.017)	(0.017)	(0.017)
Days ($\times 10^{-3}$)		-0.629***	-0.492**	-0.718***		-0.508
		(0.216)	(0.239)	(0.215)		(0.577)
Days^2 ($\times 10^{-6}$)			-0.063***	0.252***		0.389
			(0.020)	(0.046)		(0.690)
Days^3 ($\times 10^{-9}$)				-.0972***		-.206
				(.0147)		(.239)
Observations	966	966	966	966	966	966
R-square	0.572	0.575	0.596	0.627	0.665	0.669
Y-mean	6.027	6.027	6.027	6.027	6.027	6.027
Y-std.dev.	0.052	0.052	0.052	0.052	0.052	0.052
DOW FEs	Y	Y	Y	Y	Y	Y
Month FEs	Y	Y	Y	Y		
Year FEs	Y	Y	Y	Y		
Year-Month FEs					Y	Y

Notes: Standard errors are clustered at the event level. * significant 10% level; ** significant at 5% level; *** significant at 1% level.

Table S12: Reduced form, 8 events Nov 14, 2016-2017 on 2020 betting odds

	Panel A: Trump's odds					
	(1)	(2)	(3)	(4)	(5)	(6)
Holiday_RU	0.912*	0.708*	0.722**	0.475*	0.912*	0.475*
	(0.501)	(0.382)	(0.331)	(0.266)	(0.501)	(0.266)
Holiday_US	-0.542	-0.569	-0.991*	-1.033**	-0.542	-1.033**
	(0.419)	(0.400)	(0.535)	(0.475)	(0.419)	(0.475)
Days ($\times 10^{-2}$)		-6.521	-28.695	-72.052**		-72.052**
		(6.551)	(17.992)	(31.536)		(31.536)
Days^2 ($\times 10^{-4}$)			7.275*	36.350***		36.350***
			(3.908)	(14.064)		(14.064)
Days^3 ($\times 10^{-6}$)				-5.18***		-5.18***
				(1.89)		(1.89)
Observations	168	168	168	168	168	168

R-square	0.952	0.953	0.959	0.967	0.952	0.967
Y-mean	31.366	31.366	31.366	31.366	31.366	31.366
Y-std.dev.	7.066	7.066	7.066	7.066	7.066	7.066
Replications	1000	1000	1000	1000	1000	1000
	Panel B: Republican's odds					
Holiday_RU	0.863	0.175	0.195	-0.028	0.863	-0.028
	(0.617)	(0.356)	(0.330)	(0.322)	(0.617)	(0.322)
Holiday_US	0.561	0.470	-0.128	-0.166	0.561	-0.166
	(0.501)	(0.361)	(0.523)	(0.486)	(0.501)	(0.486)
Days ($\times 10^{-2}$)		-21.988***	-53.415***	-92.635**		-92.635**
		(7.780)	(20.574)	(40.289)		(40.289)
Days^2 ($\times 10^{-4}$)			10.311**	36.612**		36.612**
			(4.495)	(18.287)		(18.287)
Days^3 ($\times 10^{-6}$)				-4.69*		-4.69*
				(2.46)		(2.46)
Observations	168	168	168	168	168	168
R-square	0.840	0.870	0.886	0.895	0.840	0.895
Y-mean	45.179	45.179	45.179	45.179	45.179	45.179
Y-std.dev.	5.894	5.894	5.894	5.894	5.894	5.894
Replications	1000	1000	1000	1000	1000	1000
	Panel C: Democrat's odds					
Holiday_RU	0.034	0.029	0.024	0.110	0.034	0.110
	(0.242)	(0.385)	(0.363)	(0.325)	(0.242)	(0.325)
Holiday_US	-0.445	-0.445	-0.287	-0.272	-0.445	-0.272
	(0.425)	(0.436)	(0.745)	(0.768)	(0.425)	(0.768)
Days ($\times 10^{-2}$)		-0.143	8.188	23.367		23.367
		(10.982)	(27.270)	(34.916)		(34.916)
Days^2 ($\times 10^{-4}$)			-2.733	-12.912		-12.912
			(4.937)	(12.018)		(12.018)
Days^3 ($\times 10^{-6}$)				1.81		1.81
				(1.54)		(1.54)
Observations	168	168	168	168	168	168
R-square	0.779	0.779	0.781	0.783	0.779	0.783
Y-mean	33.193	33.193	33.193	33.193	33.193	33.193
Y-std.dev.	4.713	4.713	4.713	4.713	4.713	4.713
Replications	1000	1000	1000	1000	1000	1000
DOW FEs	Y	Y	Y	Y	Y	Y
Month FEs	Y	Y	Y	Y		
Year FEs	Y	Y	Y	Y		
Year-Month FEs					Y	Y

Notes: Bootstrap standard errors are clustered at the distinct event level. * significant 10% level; ** significant at 5% level; *** significant at 1% level.

S2.3 Reduced form for financial indexes

As financial indexes are available only on trading days, we use an unbalanced panel in Panel A and fill missing data using observation on the last trading day in Panel B. We find small and imprecise estimates on `Holiday_RU` in both panels. Estimates on `Holiday_US` are also insignificant but slightly larger.

Table S13: Reduced form, 46 events 2012-2017 on financial indexes

	Panel A			
	CBOE Volatility	Nasdaq	DJIA	S&P 500
	(1)	(2)	(3)	(4)
Holiday_RU	0.331	0.243	-3.995	-3.916
	(0.251)	(1.276)	(4.134)	(3.796)
Holiday_US	-0.828	-1.641	-4.302	3.804
	(0.846)	(8.872)	(33.693)	(26.003)
Observations	683	683	683	683
R-square	0.652	0.982	0.965	0.981
Y-mean	15.129	394.487	1678.001	1896.815
Y-std.dev.	3.424	101.335	265.725	343.042
Replications	1000	1000	1000	1000
	Panel B			
	CBOE Volatility	Nasdaq	DJIA	S&P 500
Holiday_RU	0.180	-0.114	0.533	-0.147
	(0.197)	(0.666)	(2.459)	(2.626)
Holiday_US	-0.274	-0.754	2.239	1.738
	(0.451)	(1.824)	(7.038)	(7.297)
Observations	966	966	966	966
R-square	0.610	0.979	0.961	0.978
Y-mean	15.019	397.943	1687.580	1910.560
Y-std.dev.	3.441	98.942	259.440	332.529
Replications	1000	1000	1000	1000
DOW FEs	Y	Y	Y	Y
Month FEs	Y	Y	Y	Y
Year FEs	Y	Y	Y	Y

Notes: Nasdaq and DJIA are divided by 10. Bootstrap standard errors are clustered at the distinct event level. * significant 10% level; ** significant at 5% level; *** significant at 1% level.

S2.4 Robustness of the first stage

Table S14: First stage, drop 10 busiest days, 11 days around 43, 43, 46 events 2012-2017

	Panel A: #All tweets (z)					
	(1)	(2)	(3)	(4)	(5)	(6)
Holiday_RU	-0.204***	-0.208***	-0.210***	-0.208***	-0.201***	-0.207***
	(0.070)	(0.071)	(0.071)	(0.071)	(0.072)	(0.074)
Holiday_US	-0.203	-0.225	-0.231	-0.230	-0.250**	-0.279**
	(0.157)	(0.146)	(0.152)	(0.142)	(0.118)	(0.121)
Days ($\times 10^{-3}$)		-5.187	-3.056	-7.377		-5.310
		(7.762)	(7.860)	(7.449)		(8.586)
Days^2 ($\times 10^{-6}$)			-0.861	5.060***		-0.017
			(0.563)	(1.806)		(18.298)
Days^3 ($\times 10^{-9}$)				-1.85***		-.369
				(.55)		(6.37)
Observations	473	473	473	473	473	473
R-square	0.570	0.570	0.582	0.611	0.746	0.746
Y-mean	587.6	587.6	587.6	587.6	587.6	587.6
Y-std.dev.	816.2	816.2	816.2	816.2	816.2	816.2
	Panel B: #Original tweets (z)					
Holiday_RU	-0.203**	-0.204**	-0.206**	-0.204**	-0.207**	-0.206**
	(0.075)	(0.077)	(0.077)	(0.076)	(0.080)	(0.082)
Holiday_US	-0.135	-0.140	-0.148	-0.148	-0.107	-0.107
	(0.110)	(0.112)	(0.124)	(0.125)	(0.102)	(0.098)
Days ($\times 10^{-3}$)		-1.255	1.609	-1.473		-2.529
		(6.655)	(6.699)	(7.053)		(10.529)
Days^2 ($\times 10^{-6}$)			-1.156***	3.066		-1.000
			(0.334)	(2.830)		(19.942)
Days^3 ($\times 10^{-9}$)				-1.32		1.13
				(.912)		(6.02)
Observations	473	473	473	473	473	473
R-square	0.571	0.571	0.593	0.608	0.721	0.721
Y-mean	312.3	312.3	312.3	312.3	312.3	312.3
Y-std.dev.	452.9	452.9	452.9	452.9	452.9	452.9
	Panel C: #Retweeted tweets (z)					
Holiday_RU	-0.161**	-0.163**	-0.165**	-0.162**	-0.153*	-0.160*
	(0.076)	(0.078)	(0.078)	(0.078)	(0.076)	(0.081)
Holiday_US	-0.229	-0.242	-0.236	-0.218	-0.216	-0.251*
	(0.177)	(0.173)	(0.174)	(0.170)	(0.141)	(0.149)
Days ($\times 10^{-3}$)		-3.109	-1.840	-6.740		-5.315
		(13.491)	(13.470)	(13.485)		(17.015)
Days^2 ($\times 10^{-6}$)			-0.717	5.639***		-0.326
			(0.883)	(1.760)		(37.307)

Days^3 ($\times 10^{-9}$)				-2.04***		-.765
				(.583)		(12.2)
Observations	506	506	506	506	506	506
R-square	0.425	0.425	0.432	0.471	0.684	0.685
Y-mean	226.9	226.9	226.9	226.9	226.9	226.9
Y-std.dev.	455.2	455.2	455.2	455.2	455.2	455.2
DOW FEs	Y	Y	Y	Y	Y	Y
Month FEs	Y	Y	Y	Y		
Year FEs	Y	Y	Y	Y		
Year-Month FEs					Y	Y

Notes: Standard errors are clustered at the event level. * significant 10% level; ** significant at 5% level; *** significant at 1% level.

Table S15: First stage, drop January 1 and January 7

	Panel A: #All tweets (z)					
	(1)	(2)	(3)	(4)	(5)	(6)
Y-mean	581.3	581.3	581.3	581.3	581.3	581.3
Y-std.dev.	809.6	809.6	809.6	809.6	809.6	809.6
	Panel B: #Original tweets (z)					
Y-mean	333.0	333.0	333.0	333.0	333.0	333.0
Y-std.dev.	507.6	507.6	507.6	507.6	507.6	507.6
	Panel C: #Retweeted tweets (z)					
Y-mean	236.4	236.4	236.4	236.4	236.4	236.4
Y-std.dev.	466.1	466.1	466.1	466.1	466.1	466.1
DOW FEs	Y	Y	Y	Y	Y	Y
Month FEs	Y	Y	Y	Y		
Year FEs	Y	Y	Y	Y		
Year-Month FEs					Y	Y

Notes: Standard errors are clustered at the event level. * significant 10% level; ** significant at 5% level; *** significant at 1% level.

S3 Holiday Event Study Nov 2014-Nov 2016

S3.1 First stage

Table S16: First stage, drop 10 busiest days 2012-2017, 11, 11, 15 events

	(1)	(2)	(3)	(4)	(5)	(6)
	Panel A: #All tweets (z)					
Holiday_RU	-0.606***	-0.615***	-0.615***	-0.615***	-0.613***	-0.656***
	(0.152)	(0.167)	(0.149)	(0.148)	(0.151)	(0.144)
Holiday_US	-0.297	-0.308	-0.307	-0.248	-0.310	-0.288
	(0.297)	(0.299)	(0.276)	(0.281)	(0.284)	(0.270)
Days ($\times 10^{-3}$)		-3.196	-2.006	-76.930		195.946
		(24.472)	(58.423)	(154.007)		(134.343)
Days^2 ($\times 10^{-6}$)			1.775	-248.456		440.550
			(83.335)	(420.054)		(391.056)
Days^3 ($\times 10^{-9}$)				-229		237
				(359)		(350)
Observations	231	231	231	231	231	231
R-square	0.328	0.329	0.329	0.331	0.366	0.435
Replications	1000	1000	1000	1000	1000	1000
Y-mean	1183.2	1183.2	1183.2	1183.2	1183.2	1183.2
Y-std.dev.	759.4	759.4	759.4	759.4	759.4	759.4
	Panel B: #Original tweets (z)					
Holiday_RU	-0.454***	-0.448***	-0.452***	-0.452***	-0.462***	-0.471***
	(0.106)	(0.133)	(0.148)	(0.148)	(0.107)	(0.147)
Holiday_US	-0.499***	-0.492***	-0.487***	-0.365*	-0.516***	-0.434**
	(0.137)	(0.146)	(0.148)	(0.194)	(0.132)	(0.182)
Days ($\times 10^{-3}$)		2.028	11.329	-143.665		7.190
		(17.888)	(39.521)	(148.090)		(137.396)
Days^2 ($\times 10^{-6}$)			13.877	-503.774		-75.411
			(75.011)	(442.265)		(407.898)
Days^3 ($\times 10^{-9}$)				-474		-138
				(394)		(374)
Observations	231	231	231	231	231	231
R-square	0.448	0.448	0.454	0.465	0.488	0.501
Replications	1000	1000	1000	1000	1000	1000
Y-mean	780.0	780.0	780.0	780.0	780.0	780.0
Y-std.dev.	447.3	447.3	447.3	447.3	447.3	447.3
	Panel C: #Retweeted tweets (z)					
Holiday_RU	-0.448**	-0.457**	-0.457**	-0.457**	-0.448**	-0.465**
	(0.176)	(0.202)	(0.201)	(0.194)	(0.176)	(0.181)
Holiday_US	0.109	0.091	0.093	0.092	0.107	0.068

	(0.198)	(0.228)	(0.229)	(0.217)	(0.196)	(0.202)
Days ($\times 10^{-3}$)		-4.216	-5.403	-8.832		315.498*
		(30.253)	(34.561)	(142.859)		(182.664)
Days^2 ($\times 10^{-6}$)			-1.702	-11.692		829.532
			(19.059)	(385.452)		(523.212)
Days^3 ($\times 10^{-9}$)				-7.88		592
				(289)		(387)
Observations	315	315	315	315	315	315
R-square	0.348	0.348	0.349	0.350	0.370	0.430
Replications	1000	1000	1000	1000	1000	1000
Y-mean	301.1	301.1	301.1	301.1	301.1	301.1
Y-std.dev.	513.4	513.4	513.4	513.4	513.4	513.4
DOW FEs	Y	Y	Y	Y	Y	Y
Month FEs	Y	Y	Y	Y		
Year FEs	Y	Y	Y	Y		
Year-Month FEs					Y	Y

Notes: Bootstrap standard errors are clustered at the distinct event level. * significant 10% level; ** significant at 5% level; *** significant at 1% level.

Table S17: First stage, 15 events

	Panel A: #All tweets (z)					
	(1)	(2)	(3)	(4)	(5)	(6)
Holiday_RU	-0.361***	-0.305***	-0.308***	-0.308***	-0.360***	-0.311***
	(0.101)	(0.097)	(0.095)	(0.094)	(0.098)	(0.091)
Holiday_US	-0.319	-0.217	-0.227	-0.230	-0.320	-0.163
	(0.281)	(0.315)	(0.319)	(0.314)	(0.285)	(0.314)
Days ($\times 10^{-3}$)		24.466	31.757	10.232		89.533
		(24.083)	(26.215)	(108.139)		(118.127)
Days^2 ($\times 10^{-6}$)			10.457	-52.263		273.144
			(21.250)	(306.666)		(361.278)
Days^3 ($\times 10^{-9}$)				-49.5		258
				(245)		(308)
Observations	315	315	315	315	315	315
R-square	0.157	0.170	0.207	0.210	0.219	0.245
Replications	1000	1000	1000	1000	1000	1000
Y-mean	1725.2	1725.2	1725.2	1725.2	1725.2	1725.2
Y-std.dev.	2487.6	2487.6	2487.6	2487.6	2487.6	2487.6
	Panel B: #Original tweets (z)					
Holiday_RU	-0.267***	-0.210**	-0.213**	-0.213**	-0.267***	-0.214**
	(0.102)	(0.094)	(0.093)	(0.093)	(0.100)	(0.090)
Holiday_US	-0.341	-0.235	-0.245	-0.248	-0.340	-0.176

	(0.288)	(0.320)	(0.323)	(0.321)	(0.292)	(0.318)
Days ($\times 10^{-3}$)		25.252	32.764	12.015		24.337
		(23.255)	(26.461)	(102.663)		(107.828)
Days^2 ($\times 10^{-6}$)			10.772	-49.685		101.603
			(22.235)	(288.580)		(331.536)
Days^3 ($\times 10^{-9}$)				-47.7		136
				(229)		(285)
Observations	315	315	315	315	315	315
R-square	0.194	0.208	0.247	0.251	0.256	0.286
Replications	1000	1000	1000	1000	1000	1000
Y-mean	1424.1	1424.1	1424.1	1424.1	1424.1	1424.1
Y-std.dev.	2495.2	2495.2	2495.2	2495.2	2495.2	2495.2
	Panel C: #Retweeted tweets (z)					
Holiday_RU	-0.448**	-0.457**	-0.457**	-0.457**	-0.448**	-0.465**
	(0.176)	(0.202)	(0.201)	(0.194)	(0.176)	(0.181)
Holiday_US	0.109	0.091	0.093	0.092	0.107	0.068
	(0.198)	(0.228)	(0.229)	(0.217)	(0.196)	(0.202)
Days ($\times 10^{-3}$)		-4.216	-5.403	-8.832		315.498*
		(30.253)	(34.561)	(142.859)		(182.664)
Days^2 ($\times 10^{-6}$)			-1.702	-11.692		829.532
			(19.059)	(385.452)		(523.212)
Days^3 ($\times 10^{-9}$)				-7.88		592
				(289)		(387)
Observations	315	315	315	315	315	315
R-square	0.348	0.348	0.349	0.350	0.370	0.430
Replications	1000	1000	1000	1000	1000	1000
Y-mean	301.1	301.1	301.1	301.1	301.1	301.1
Y-std.dev.	513.4	513.4	513.4	513.4	513.4	513.4
DOW FEs	Y	Y	Y	Y	Y	Y
Month FEs	Y	Y	Y	Y		
Year FEs	Y	Y	Y	Y		
Year-Month FEs					Y	Y

Notes: Bootstrap standard errors are clustered at the distinct event level. * significant 10% level; ** significant at 5% level; *** significant at 1% level.

S3.2 Reduced form for Iowa 2016 election odds

Table S18: Reduced form, 13 events

	(1)	(2)	(3)	(4)	(5)	(6)
	Panel A: Republican's price					
Holiday_RU	-0.011**	-0.011**	-0.011**	-0.011**	-0.011**	-0.011***
	(0.006)	(0.005)	(0.005)	(0.005)	(0.005)	(0.004)
Holiday_US	0.012*	0.012*	0.012*	0.012*	0.011**	0.012**
	(0.006)	(0.007)	(0.007)	(0.007)	(0.006)	(0.006)
Days ($\times 10^{-2}$)		-0.002	0.014	0.075		-0.033
		(0.083)	(0.083)	(0.177)		(0.134)
Days^2 ($\times 10^{-4}$)			-0.002	-0.031		0.031
			(0.004)	(0.074)		(0.053)
Days^3 ($\times 10^{-6}$)				.00308		-.00377
				(.0087)		(.0056)
Observations	273	273	273	273	273	273
R-square	0.904	0.904	0.907	0.910	0.919	0.919
Y-mean	0.399	0.399	0.399	0.399	0.399	0.399
Y-std.dev.	0.069	0.069	0.069	0.069	0.069	0.069
Replications	1000	1000	1000	1000	1000	1000
	Panel B: Democrat's price					
Holiday_RU	0.015***	0.016***	0.016***	0.016***	0.016***	0.016***
	(0.005)	(0.005)	(0.005)	(0.005)	(0.004)	(0.004)
Holiday_US	-0.015**	-0.013*	-0.014*	-0.014*	-0.013**	-0.014**
	(0.007)	(0.007)	(0.007)	(0.007)	(0.007)	(0.006)
Days ($\times 10^{-2}$)		0.023	0.003	-0.093		-0.005
		(0.084)	(0.087)	(0.181)		(0.137)
Days^2 ($\times 10^{-4}$)			0.002	0.049		-0.011
			(0.005)	(0.076)		(0.055)
Days^3 ($\times 10^{-6}$)				-.00489		.00205
				(.00894)		(.00584)
Observations	273	273	273	273	273	273
R-square	0.893	0.893	0.897	0.904	0.914	0.915
Y-mean	0.608	0.608	0.608	0.608	0.608	0.608
Y-std.dev.	0.071	0.071	0.071	0.071	0.071	0.071
Replications	1000	1000	1000	1000	1000	1000
DOW FEs	Y	Y	Y	Y	Y	Y
Month FEs	Y	Y	Y	Y		
Year FEs	Y	Y	Y	Y		
Year-Month FEs					Y	Y

Notes: Betting data is missing on Nov 1-Nov 2 2015 and Jan 16-Jan 18 2016, so #events is smaller than that in Table S17 by 2. Bootstrap standard errors are clustered at the distinct event level. * significant 10% level; ** significant at 5% level; *** significant at 1% level.

Table S19: Reduced form, 15 events

	(1)	(2)	(3)	(4)	(5)	(6)
	Panel A: Republican's price					
Holiday_RU	-0.010**	-0.010**	-0.010**	-0.010**	-0.010**	-0.010**
	(0.005)	(0.004)	(0.004)	(0.005)	(0.004)	(0.004)
Holiday_US	0.008	0.008	0.008	0.008	0.007	0.008
	(0.005)	(0.006)	(0.006)	(0.006)	(0.005)	(0.005)
Days ($\times 10^{-2}$)		0.002	0.020	0.078		-0.029
		(0.072)	(0.073)	(0.161)		(0.128)
Days^2 ($\times 10^{-4}$)			-0.002	-0.031		0.027
			(0.005)	(0.068)		(0.050)
Days^3 ($\times 10^{-6}$)				.00306		-.00326
				(.00814)		(.00582)
Observations	315	315	315	315	315	315
R-square	0.900	0.900	0.904	0.908	0.917	0.917
Y-mean	0.398	0.398	0.398	0.398	0.398	0.398
Y-std.dev.	0.064	0.064	0.064	0.064	0.064	0.064
Replications	1000	1000	1000	1000	1000	1000
	Panel B: Democrat's price					
Holiday_RU	0.015***	0.015***	0.015***	0.015***	0.015***	0.015***
	(0.004)	(0.004)	(0.004)	(0.004)	(0.003)	(0.004)
Holiday_US	-0.008	-0.008	-0.009	-0.009	-0.008	-0.008
	(0.006)	(0.006)	(0.006)	(0.006)	(0.006)	(0.006)
Days ($\times 10^{-2}$)		0.001	-0.024	-0.115		0.064
		(0.075)	(0.077)	(0.170)		(0.136)
Days^2 ($\times 10^{-4}$)			0.003	0.048		-0.047
			(0.005)	(0.075)		(0.055)
Days^3 ($\times 10^{-6}$)				-.00485		.00561
				(.00893)		(.00616)
Observations	315	315	315	315	315	315
R-square	0.879	0.879	0.887	0.895	0.906	0.907
Y-mean	0.609	0.609	0.609	0.609	0.609	0.609
Y-std.dev.	0.067	0.067	0.067	0.067	0.067	0.067
Replications	1000	1000	1000	1000	1000	1000
DOW FEs	Y	Y	Y	Y	Y	Y
Month FEs	Y	Y	Y	Y		
Year FEs	Y	Y	Y	Y		
Year-Month FEs					Y	Y

Notes: We filled missing bet price on Nov 1-Nov 2 2015 and Jan 16-Jan 18 2016 with the last available price before the missing day, namely price on Oct 31 2015 and Jan 15 2016. Bootstrap standard errors are clustered at the distinct event level. * significant 10% level; ** significant at 5% level; *** significant at 1% level.

Figure S2: Event Study of Iowa betting odds around Russian holidays Nov 2014-Nov 2016

(Betting data is missing on Nov 1-Nov 2 2015 and Jan 16-Jan 18 2016, so #events is smaller by 2.)

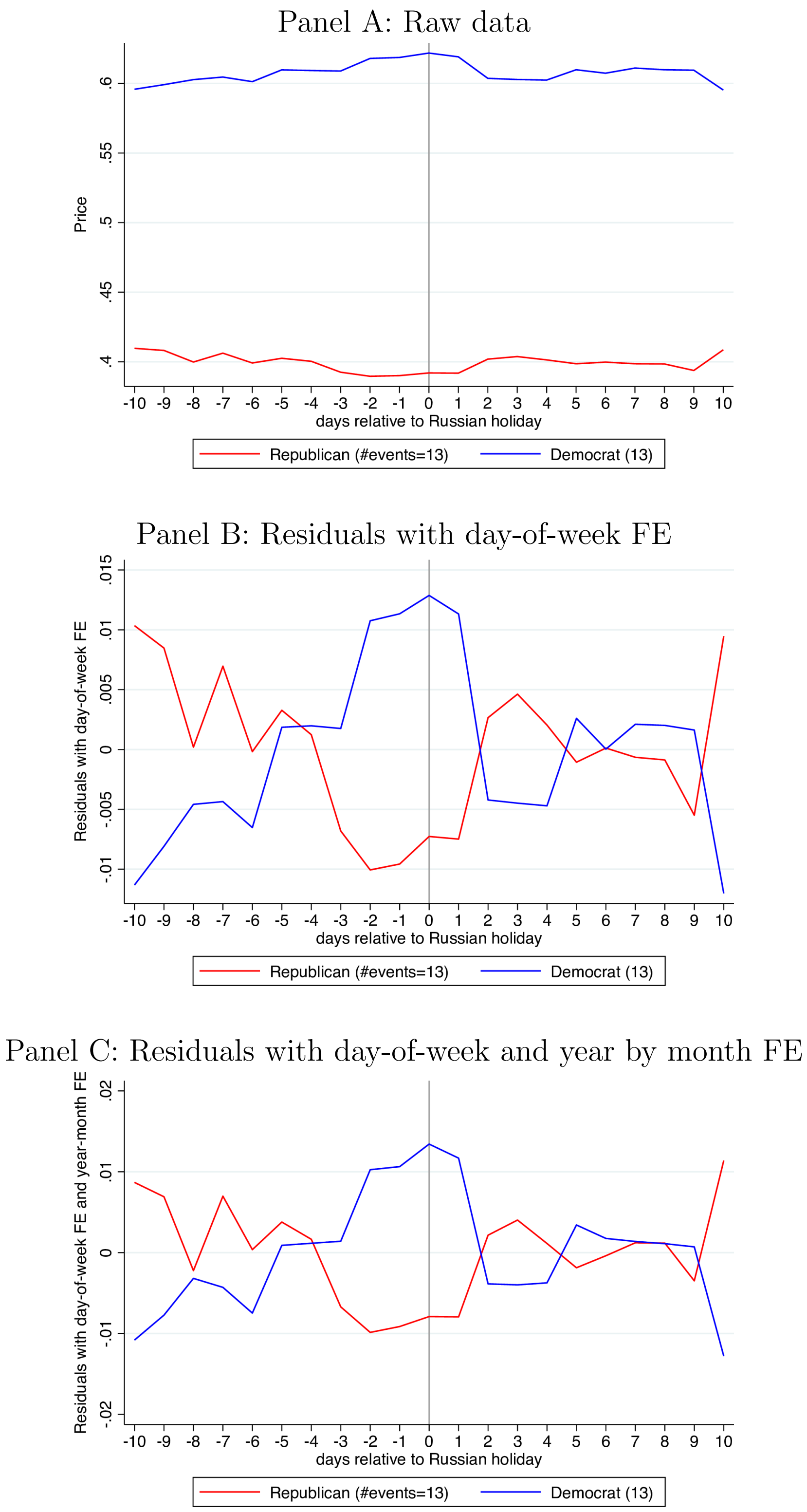

S4 Regression Discontinuity Estimates of First Stage

As noted in the main text, Twitter announced suspicious Russian accounts in four separate waves. The announcement date is thought to follow the blocking date with a substantial lag. Unfortunately, Twitter does not disclose the date at which it blocked these suspicious tweets and accounts. Nevertheless, we can try and infer major blocking dates by discontinuous reductions in trolling activity.[1] Table S20 uses the reduction in tweets following May 6, 2017 as a threshold in an Regression Discontinuity Design analysis. Depending on the specification, tweets fall by 1,600-2,500 tweets per day after May 6, 2017. This is driven by a 1,600-2,000 reduction in retweets, with original tweets showing a less consistent response. We do not pursue this RD analysis further because:

1. As RD analyses go, this is not a particularly robust first stage estimate.
2. Twitter does not report May 6, 2017 as a major blocking date, we infer it.
3. Particularly given the previous point, other factors might drive some of the rapid eduction in trolling activity after May 6, 2017 that would not be exogenous to the Hedonometer or President Trump's re-election odds.

Without additional information on blocking dates, we favor identifying variation in trolling from Russian holidays and ambient temperature in St. Petersburg.

Table S20: Wave 1, May 6, 2017 as cutoff

	Panel A: #All tweets ($\times 10^{-2}$)			
	(1)	(2)	(3)	(4)
Post	-21.347***	-21.948***	-25.478***	-15.919***
	(0.838)	(1.338)	(0.963)	(1.357)
Relative days ($\times 10^{-2}$)		1.112		-4.192**
		(2.229)		(2.014)
Post $\times$ Relative days ($\times 10^{-2}$)		-1.188		-4.222*
		(2.879)		(2.168)
Observations	230	230	304	304
R-square	0.740	0.740	0.699	0.735
Y-mean	24.717	24.717	23.978	23.978
Y-std.dev.	12.436	12.436	15.266	15.266
	Panel B: #Original tweets ($\times 10^{-2}$)			
Post	-5.383***	-0.219	-5.383***	-0.219
	(0.474)	(0.605)	(0.474)	(0.605)
Relative days ($\times 10^{-2}$)		-0.583		-0.583
		(0.617)		(0.617)

[1] We thank Joe Doyle (MIT) for a conversation that spurred this exercise.

Post × Relative days (×10^{-2})		-5.666***		-5.666***
		(0.830)		(0.830)
Observations	304	304	304	304
R-square	0.299	0.456	0.299	0.456
Y-mean	10.405	10.405	10.405	10.405
Y-std.dev.	4.929	4.929	4.929	4.929
	Panel C: #Retweeted tweets (×10^{-2})			
Post	-18.088***	-19.143***	-20.094***	-15.700***
	(0.548)	(0.855)	(0.685)	(1.062)
Relative days (×10^{-2})		1.869		-3.608**
		(1.349)		(1.720)
Post × Relative days (×10^{-2})		-2.053		1.444
		(1.496)		(1.757)
Observations	248	248	304	304
R-square	0.816	0.818	0.740	0.753
Y-mean	13.208	13.208	13.573	13.573
Y-std.dev.	10.032	10.032	11.698	11.698

Notes: We use CCT and IK bandwidths in Column (1)-(2) and Column (3)-(4). Robust standard errors are reported in parentheses. * significant 10% level; ** significant at 5% level; *** significant at 1% level.

S5 Hedonometer's Response to US Events

Table S21: Hedonometer's responses to other events

	Hedonometer					
	(1)	(2)	(3)	(4)	(5)	(6)
Brett Kavanaugh	-0.063**	-0.061**	-0.018	-0.007	0.017	-0.014
	(0.030)	(0.030)	(0.030)	(0.030)	(0.030)	(0.030)
Holiday_US	0.056***	0.056***	0.056***	0.056***	0.056***	0.056***
	(0.004)	(0.004)	(0.004)	(0.004)	(0.004)	(0.004)
Observations	2922	2922	2922	2922	2922	2922
R-square	0.616	0.616	0.616	0.616	0.616	0.616
Dayton shooting	-0.206***	-0.116***	-0.054*	-0.044	-0.022	-0.010
	(0.030)	(0.030)	(0.030)	(0.030)	(0.030)	(0.030)
Holiday_US	0.056***	0.056***	0.056***	0.056***	0.056***	0.056***
	(0.004)	(0.004)	(0.004)	(0.004)	(0.004)	(0.004)
Observations	2922	2922	2922	2922	2922	2922
R-square	0.622	0.617	0.616	0.616	0.616	0.616
DOW FEs	Y	Y	Y	Y	Y	Y
Month FEs	Y	Y	Y	Y		
Year FEs	Y	Y	Y	Y		
Year-Month FEs					Y	Y

Notes: * significant 10% level; ** significant at 5% level; *** significant at 1% level.

S6 Robustness with Blank Tweets

Table S22: First stage: Russian holiday and temperature on blocked tweets on the day shift

	Panel A: #All tweets (z)					
	(1)	(2)	(3)	(4)	(5)	(6)
Holiday_RU × Wave=1	-0.339***	-0.339***	-0.338***	-0.338***	-0.340***	-0.341***
	(0.103)	(0.103)	(0.103)	(0.103)	(0.092)	(0.093)
Holiday_RU × Wave=2	-0.040	-0.039	-0.039	-0.039	-0.039	-0.039
	(0.103)	(0.103)	(0.103)	(0.103)	(0.092)	(0.093)
Holiday_RU × Wave=3	-0.005	-0.004	-0.004	-0.004	-0.005	-0.005
	(0.103)	(0.103)	(0.103)	(0.103)	(0.092)	(0.093)
Holiday_RU × Wave=4	-0.007	-0.006	-0.006	-0.006	-0.007	-0.007
	(0.103)	(0.103)	(0.103)	(0.103)	(0.092)	(0.093)
Temperature × Wave=1 (z)	.0567*	.0567*	.0558*	.0547*	.00155	.0015
	(.0323)	(.0323)	(.0323)	(.0323)	(.0332)	(.0332)
Temperature × Wave=2 (z)	-.0147	-.0147	-.0157	-.0167	.00445	.0044
	(.0323)	(.0323)	(.0323)	(.0323)	(.0332)	(.0332)
Temperature × Wave=3 (z)	.00114	.00114	.000213	-.000858	.0017	.00164
	(.0323)	(.0323)	(.0323)	(.0323)	(.0332)	(.0332)
Temperature × Wave=4 (z)	-.000878	-.000881	-.0018	-.00287	.000348	.000295
	(.0323)	(.0323)	(.0323)	(.0323)	(.0332)	(.0332)
Holiday_US	.013	.0131	.0128	.013	.0139	.014
	(.0474)	(.0474)	(.0474)	(.0474)	(.0426)	(.0426)
Days ($\times 10^{-3}$)		.0916	.616	.849		-.0479
		(.853)	(.861)	(.876)		(2.28)
Days^2 ($\times 10^{-6}$)			-.186***	-.425**		.307
			(.0421)	(.172)		(1.8)
Days^3 ($\times 10^{-9}$)				.0547		-.103
				(.0382)		(.398)
Observations	11560	11560	11560	11560	11560	11560
R-square	0.363	0.363	0.364	0.364	0.501	0.501
Y-mean	167.3	167.3	167.3	167.3	167.3	167.3
Y-std.dev.	689.1	689.1	689.1	689.1	689.1	689.1
Y-mean Wave1	569.9	569.9	569.9	569.9	569.9	569.9
Y-std.dev. Wave1	1285.5	1285.5	1285.5	1285.5	1285.5	1285.5
Y-mean Wave2	93.30	93.30	93.30	93.30	93.30	93.30
Y-std.dev. Wave2	159.3	159.3	159.3	159.3	159.3	159.3
Y-mean Wave3	0.0010	0.0010	0.0010	0.0010	0.0010	0.0010
Y-std.dev. Wave3	0.0416	0.0416	0.0416	0.0416	0.0416	0.0416
Y-mean Wave4	5.816	5.816	5.816	5.816	5.816	5.816
Y-std.dev. Wave4	19.39	19.39	19.39	19.39	19.39	19.39

	Panel B: #Original tweets (z)					
	(1)	(2)	(3)	(4)	(5)	(6)
Holiday_RU × Wave=1	-0.316***	-0.316***	-0.316***	-0.315***	-0.317***	-0.317***
	(0.109)	(0.110)	(0.110)	(0.110)	(0.100)	(0.100)
Holiday_RU × Wave=2	-0.023	-0.022	-0.022	-0.022	-0.023	-0.023
	(0.109)	(0.110)	(0.110)	(0.110)	(0.100)	(0.100)
Holiday_RU × Wave=3	-0.005	-0.005	-0.005	-0.005	-0.006	-0.006
	(0.109)	(0.110)	(0.110)	(0.110)	(0.100)	(0.100)
Holiday_RU × Wave=4	-0.007	-0.007	-0.007	-0.007	-0.008	-0.008
	(0.109)	(0.110)	(0.110)	(0.110)	(0.100)	(0.100)
Temperature × Wave=1 (z)	.099***	.099***	.0984***	.0948***	.0326	.0326
	(.0343)	(.0343)	(.0343)	(.0343)	(.036)	(.036)
Temperature × Wave=2 (z)	.00842	.00841	.00787	.00424	.00992	.00991
	(.0343)	(.0343)	(.0343)	(.0343)	(.036)	(.036)
Temperature × Wave=3 (z)	.000473	.000471	-.0000732	-.0037	.000829	.000813
	(.0343)	(.0343)	(.0343)	(.0343)	(.036)	(.036)
Temperature × Wave=4 (z)	-.00192	-.00192	-.00246	-.00609	-.000883	-.0009
	(.0343)	(.0343)	(.0343)	(.0343)	(.036)	(.036)
Holiday_US	.0303	.0304	.0302	.0308	.0327	.0327
	(.0504)	(.0504)	(.0504)	(.0504)	(.0462)	(.0462)
Days ($\times 10^{-3}$)		.0598	.369	1.16		.133
		(.907)	(.916)	(.931)		(2.47)
Days^2 ($\times 10^{-6}$)			-.11**	-.921***		.0604
			(.0448)	(.183)		(1.95)
Days^3 ($\times 10^{-9}$)				.185***		-.0359
				(.0406)		(.432)
Observations	11560	11560	11560	11560	11560	11560
R-square	0.277	0.277	0.278	0.279	0.410	0.410
Y-mean	94.01	94.01	94.01	94.01	94.01	94.01
Y-std.dev.	560.6	560.6	560.6	560.6	560.6	560.6
Y-mean Wave1	356.8	356.8	356.8	356.8	356.8	356.8
Y-std.dev. Wave1	1078.0	1078.0	1078.0	1078.0	1078.0	1078.0
Y-mean Wave2	15.47	15.47	15.47	15.47	15.47	15.47
Y-std.dev. Wave2	55.23	55.23	55.23	55.23	55.23	55.23
Y-mean Wave3	0.0010	0.0010	0.0010	0.0010	0.0010	0.0010
Y-std.dev. Wave3	0.0416	0.0416	0.0416	0.0416	0.0416	0.0416
Y-mean Wave4	4.401	4.401	4.401	4.401	4.401	4.401
Y-std.dev. Wave4	18.92	18.92	18.92	18.92	18.92	18.92

	Panel C: #Retweeted tweets (z)					
	(1)	(2)	(3)	(4)	(5)	(6)
Holiday_RU × Wave=1	-0.185*	-0.184*	-0.184*	-0.184*	-0.186**	-0.186**
	(0.102)	(0.102)	(0.102)	(0.102)	(0.084)	(0.084)
Holiday_RU × Wave=2	-0.047	-0.047	-0.047	-0.047	-0.046	-0.046
	(0.102)	(0.102)	(0.102)	(0.102)	(0.084)	(0.084)
Holiday_RU × Wave=3	-0.000	0.000	0.001	0.001	0.000	-0.000
	(0.102)	(0.102)	(0.102)	(0.102)	(0.084)	(0.084)
Holiday_RU × Wave=4	-0.002	-0.001	-0.001	-0.001	-0.001	-0.002
	(0.102)	(0.102)	(0.102)	(0.102)	(0.084)	(0.084)
Temperature × Wave=1 (z)	-.054*	-.054*	-.0551*	-.0508	-.0565*	-.0566*
	(.032)	(.032)	(.032)	(.032)	(.03)	(.03)
Temperature × Wave=2 (z)	-.0488	-.0488	-.0498	-.0456	-.0082	-.00829
	(.032)	(.032)	(.032)	(.032)	(.03)	(.03)
Temperature × Wave=3 (z)	.0017	.0017	.000615	.00487	.00231	.00221
	(.032)	(.032)	(.032)	(.032)	(.03)	(.03)
Temperature × Wave=4 (z)	.00154	.00154	.000458	.00471	.00241	.00232
	(.032)	(.032)	(.032)	(.032)	(.03)	(.03)
Holiday_US	-.0264	-.0264	-.0266	-.0274	-.0286	-.0286
	(.0471)	(.0471)	(.047)	(.047)	(.0386)	(.0386)
Days ($\times 10^{-3}$)		.0969	.713	-.213		-.354
		(.847)	(.854)	(.868)		(2.06)
Days^2 ($\times 10^{-6}$)			-.218***	.734***		.582
			(.0418)	(.171)		(1.63)
Days^3 ($\times 10^{-9}$)				-.218***		-.166
				(.0378)		(.36)
Observations	11560	11560	11560	11560	11560	11560
R-square	0.378	0.378	0.379	0.381	0.593	0.593
Y-mean	73.25	73.25	73.25	73.25	73.25	73.25
Y-std.dev.	306.4	306.4	306.4	306.4	306.4	306.4
Y-mean Wave1	213.1	213.1	213.1	213.1	213.1	213.1
Y-std.dev. Wave1	571.1	571.1	571.1	571.1	571.1	571.1
Y-mean Wave2	78.47	78.47	78.47	78.47	78.47	78.47
Y-std.dev. Wave2	139.2	139.2	139.2	139.2	139.2	139.2
Y-mean Wave3	0	0	0	0	0	0
0 Y-std.dev. Wave3	0	0	0	0	0	0
0 Y-mean Wave4	1.415	1.415	1.415	1.415	1.415	1.415
Y-std.dev. Wave4	2.761	2.761	2.761	2.761	2.761	2.761
DOW FEs	Y	Y	Y	Y	Y	Y
Wave-Month FEs	Y	Y	Y	Y		
Wave-Year FEs	Y	Y	Y	Y		
Wave-Year-Month FEs					Y	Y

Notes: The smaller sample size than four times #days 2012-2019 is due to 32 days with no temperature data. * significant 10% level; ** significant at 5% level; *** significant at 1% level.

Table S23: First stage, drop 10 busiest days, 42, 42, 44 events 2012-2017

	Panel A: #All tweets (z)					
	(1)	(2)	(3)	(4)	(5)	(6)
Holiday_RU	-0.223***	-0.235***	-0.235***	-0.233***	-0.220***	-0.234***
	(0.071)	(0.072)	(0.071)	(0.071)	(0.070)	(0.072)
Holiday_US	-0.092	-0.099	-0.101	-0.110	-0.125	-0.134
	(0.101)	(0.103)	(0.099)	(0.096)	(0.108)	(0.109)
Days ($\times 10^{-3}$)		-4.398	-2.673	-7.433		-4.049
		(6.698)	(6.748)	(6.494)		(9.571)
Days^2 ($\times 10^{-6}$)			-0.783	5.852***		-1.142
			(0.539)	(2.014)		(18.899)
Days^3 ($\times 10^{-9}$)				-2.03***		.306
				(.609)		(5.84)
Observations	882	882	882	882	882	882
R-square	0.527	0.527	0.537	0.568	0.704	0.705
Y-mean	607.4	607.4	607.4	607.4	607.4	607.4
Y-std.dev.	842.5	842.5	842.5	842.5	842.5	842.5
	Panel B: #Original tweets (z)					
Holiday_RU	-0.208***	-0.211***	-0.211***	-0.209***	-0.208***	-0.209**
	(0.071)	(0.077)	(0.077)	(0.076)	(0.071)	(0.078)
Holiday_US	-0.025	-0.027	-0.030	-0.036	-0.029	-0.033
	(0.114)	(0.113)	(0.110)	(0.107)	(0.112)	(0.110)
Days ($\times 10^{-3}$)		-1.008	1.749	-1.291		-2.346
		(4.919)	(4.838)	(5.435)		(7.922)
Days^2 ($\times 10^{-6}$)			-1.252***	2.987		-1.654
			(0.367)	(3.429)		(16.244)
Days^3 ($\times 10^{-9}$)				-1.3		1.14
				(1.09)		(4.96)
Observations	882	882	882	882	882	882
R-square	0.482	0.482	0.507	0.520	0.647	0.648
Y-mean	301.6	301.6	301.6	301.6	301.6	301.6
Y-std.dev.	466.8	466.8	466.8	466.8	466.8	466.8
	Panel C: #Retweeted tweets (z)					
Holiday_RU	-0.165*	-0.183*	-0.183*	-0.181*	-0.167*	-0.185*
	(0.090)	(0.097)	(0.097)	(0.097)	(0.090)	(0.098)
Holiday_US	-0.057	-0.073	-0.073	-0.067	-0.061	-0.072
	(0.085)	(0.087)	(0.088)	(0.087)	(0.089)	(0.094)
Days ($\times 10^{-3}$)		-6.681	-5.899	-9.686		-4.857
		(12.392)	(12.289)	(12.311)		(17.251)
Days^2 ($\times 10^{-6}$)			-0.365	5.457***		2.154
			(0.837)	(1.755)		(34.344)
Days^3 ($\times 10^{-9}$)				-1.87***		-1.6
				(.605)		(10.5)

Observations	924	924	924	924	924	924
R-square	0.373	0.374	0.376	0.404	0.626	0.627
Y-mean	224.5	224.5	224.5	224.5	224.5	224.5
Y-std.dev.	467.3	467.3	467.3	467.3	467.3	467.3
DOW FEs	Y	Y	Y	Y	Y	Y
Month FEs	Y	Y	Y	Y		
Year FEs	Y	Y	Y	Y		
Year-Month FEs					Y	Y

Notes: Standard errors are clustered at the event level. * significant 10% level; ** significant at 5% level; *** significant at 1% level.

Table S24: First stage, drop 10 busiest days 2012-2017, 11, 11, 15 events

	Panel A: #All tweets (z)					
	(1)	(2)	(3)	(4)	(5)	(6)
Holiday_RU	-0.607***	-0.615***	-0.616***	-0.616***	-0.614***	-0.657***
	(0.152)	(0.167)	(0.149)	(0.148)	(0.151)	(0.144)
Holiday_US	-0.297	-0.307	-0.307	-0.249	-0.310	-0.289
	(0.296)	(0.299)	(0.276)	(0.281)	(0.284)	(0.270)
Days ($\times 10^{-3}$)		-3.066	-1.866	-75.881		196.623
		(24.467)	(58.363)	(153.957)		(134.539)
Days^2 ($\times 10^{-6}$)			1.790	-245.408		442.604
			(83.241)	(420.179)		(391.707)
Days^3 ($\times 10^{-9}$)				-226		239
				(360)		(351)
Observations	231	231	231	231	231	231
R-square	0.328	0.329	0.329	0.331	0.366	0.435
Replications	1000	1000	1000	1000	1000	1000
Y-mean	1186.3	1186.3	1186.3	1186.3	1186.3	1186.3
Y-std.dev.	762.2	762.2	762.2	762.2	762.2	762.2
	Panel B: #Original tweets (z)					
Holiday_RU	-0.441***	-0.435***	-0.438***	-0.438***	-0.450***	-0.455***
	(0.103)	(0.132)	(0.149)	(0.148)	(0.103)	(0.147)
Holiday_US	-0.496***	-0.488***	-0.482***	-0.360*	-0.512***	-0.428**
	(0.118)	(0.132)	(0.137)	(0.188)	(0.116)	(0.175)
Days ($\times 10^{-3}$)		2.363	11.388	-145.132		-5.931
		(18.667)	(39.727)	(155.739)		(147.600)
Days^2 ($\times 10^{-6}$)			13.467	-509.284		-108.579
			(76.842)	(464.522)		(436.592)
Days^3 ($\times 10^{-9}$)				-479		-159
				(415)		(399)
Observations	231	231	231	231	231	231

R-square	0.465	0.466	0.470	0.482	0.503	0.514
Replications	1000	1000	1000	1000	1000	1000
Y-mean	759.6	759.6	759.6	759.6	759.6	759.6
Y-std.dev.	444.7	444.7	444.7	444.7	444.7	444.7
	Panel C: #Retweeted tweets (z)					
Holiday_RU	-0.411**	-0.418*	-0.418*	-0.418**	-0.411**	-0.426**
	(0.193)	(0.220)	(0.219)	(0.213)	(0.193)	(0.197)
Holiday_US	0.094	0.081	0.083	0.083	0.093	0.052
	(0.203)	(0.234)	(0.234)	(0.221)	(0.201)	(0.207)
Days ($\times 10^{-3}$)		-2.925	-3.869	-6.177		310.826*
		(31.340)	(35.328)	(142.309)		(182.049)
Days^2 ($\times 10^{-6}$)			-1.376	-8.104		811.238
			(18.901)	(383.685)		(520.717)
Days^3 ($\times 10^{-9}$)				-5.31		576
				(288)		(385)
Observations	315	315	315	315	315	315
R-square	0.347	0.347	0.348	0.348	0.367	0.426
Replications	1000	1000	1000	1000	1000	1000
Y-mean	318.6	318.6	318.6	318.6	318.6	318.6
Y-std.dev.	541.5	541.5	541.5	541.5	541.5	541.5
DOW FEs	Y	Y	Y	Y	Y	Y
Month FEs	Y	Y	Y	Y		
Year FEs	Y	Y	Y	Y		
Year-Month FEs					Y	Y

Notes: Bootstrap standard errors are clustered at the distinct event level. * significant 10% level; ** significant at 5% level; *** significant at 1% level.

S7 Examples of Twitter-identified Troll Tweets

“We have over 40 million legal immigrants in our today. Why should we accept your criminals? #IllegalAmigoz” 2015-02-18 17:39 (UTC)

“We’ve already had 8 years of the disastrous Obama presidency. Enjoy 8 years of President Trump! #DrainTheSwamp #MAGA” 2017-07-20 01:23

“RT @WalshFreedom: Thought you might like this reminder: almost a year ago, #BLM protestors in TX calling for white Cops to be killed.” 2016-07-09 00:04
